# Biomechanical evaluation of a novel repair strategy for intervertebral disc herniation in an ovine lumbar spine model

**DOI:** 10.3389/fbioe.2022.1018257

**Published:** 2022-10-25

**Authors:** Mitchell I. Page, Jeremiah T. Easley, Andres F. Bonilla, Vikas V. Patel, Christian M. Puttlitz

**Affiliations:** ^1^ Orthopaedic Bioengineering Research Laboratory, Department of Mechanical Engineering and School of Biomedical Engineering, Colorado State University, Fort Collins, CO, United States; ^2^ Preclinical Surgical Research Laboratory, C. Wayne McIlwraith Translational Medicine Institute, Department of Clinical Sciences, Colorado State University, Fort Collins, CO, United States; ^3^ Department of Orthopedics, University of Colorado, Anschutz Medical Campus, Aurora, CO, United States

**Keywords:** intervertebral disc, tissue engineering, annulus fibrosus, disc herniation, additive manufacturing

## Abstract

Following herniation of the intervertebral disc, there is a need for advanced surgical strategies to protect the diseased tissue from further herniation and to minimize further degeneration. Accordingly, a novel tissue engineered implant for annulus fibrosus (AF) repair was fabricated *via* three-dimensional fiber deposition and evaluated in a large animal model. Specifically, lumbar spine kinetics were assessed for eight (n = 8) cadaveric ovine lumbar spines in three pure moment loading settings (flexion-extension, lateral bending, and axial rotation) and three clinical conditions (intact, with a defect in the AF, and with the defect treated using the AF repair implant). In *ex vivo* testing, seven of the fifteen evaluated biomechanical measures were significantly altered by the defect. In each of these cases, the treated spine more closely approximated the intact biomechanics and four of these cases were also significantly different to the defect. The same spinal kinetics were also assessed in a preliminary *in vivo* study of three (n = 3) ovine lumbar spines 12 weeks post-implantation. Similar to the *ex vivo* results, functional efficacy of the treatment was demonstrated as compared to the defect model at 12 weeks post-implantation. These promising results motivate a future large animal study cohort which will establish statistical power of these results further elucidate the observed outcomes, and provide a platform for clinical translation of this novel AF repair patch strategy. Ultimately, the developed approach to AF repair holds the potential to maintain the long-term biomechanical function of the spine and prevent symptomatic re-herniation.

## 1 Introduction

There is a need for advanced treatment strategies to mitigate the severe pain and physical impairment associated with intervertebral disc (IVD) herniation (Leung et al., 2006; O’Halloran and Pandit, 2007; Richardson et al., 2007; Kalson et al., 2008; Sakai, 2008; Bron et al., 2009; Guterl et al., 2013). Herniation of the IVD is a leading cause of chronic low back pain, one of the most prominent and burdensome conditions in society (Heliövaara et al., 1989; Andersson, 1998; Dagenais et al., 2008). In severe cases, surgical interventions are required to treat IVD herniation, such as laminectomy with partial discectomy (Gray et al., 2006), lumbar interbody fusion (Phillips et al., 2013), or IVD arthroplasty (Hedman et al., 1991; Lee et al., 1991; Guyer and Ohnmeiss, 2003; Abi-Hanna et al., 2018). However, these current methods are palliative and do not attempt to repair or regenerate the annulus fibrosus (AF), the fibrocartilaginous outer wall of the IVD. The AF has a highly-organized, collagen-reinforced structure with multiple concentric lamellae. This structural organization produces the AF’s anisotropic and complex mechanical properties, which are essential to the proper functioning of the IVD’s mechanical role in the spinal column (Humzah and Soames, 1988; Cassidy et al., 1989; Elliott and Setton, 2001; Wagner and Lotz, 2004). Alterations in the AF’s structure are hallmarks of IVD degeneration. Accordingly, strategies that fail to restore the mechanical function of the AF often lead to symptomatic re-herniation and/or revision surgeries (Harper and Klineberg, 2019). Tissue engineering of the annulus fibrosus (AF) has the potential to arrest the IVD degeneration cascade, repair and/or regenerate the diseased tissue, and restore the complex mechanical as well as biological functions of the IVD following herniation.

A variety of novel, tissue engineering strategies and biomaterials have been proposed for AF regeneration (Nerurkar et al., 2007; Chan and Leong, 2008; Nesti et al., 2008; Koepsell et al., 2011; Park et al., 2011; Driscoll et al., 2013; Kang et al., 2013; Pirvu et al., 2015; McGuire et al., 2017). However, implementation of engineered biomaterials to an effective IVD repair strategy remains elusive. Current approaches prevalently leverage additive manufacturing methods to generate precise, organized scaffold architectures. The mechanical efficacy of these tissue engineered scaffolds is critical to afford essential structural support, functional performance, resilience to implant failure, and a micromechanical environment conducive for the generation and maintenance of the intended mature tissue (Hutmacher and Williams, 2000; Ghosh and Ingber, 2007). Biomimetic fibrous composite scaffolds with structural fibers that replicate the native collagen architecture are well suited to AF repair and have demonstrated some *in vitro* success (Nerurkar et al., 2009; Strange et al., 2014). To augment the biological relevance of small-scale fibers with the manufacturability and mechanical performance of larger fibers, a multi-scale fibrous scaffolds can be leveraged. For example, precise architectures can be printed with relatively large fibers (approximately 100–500 µm in diameter) *via* three-dimensional fiber deposition (3DF) (Woodfield et al., 2004; Moroni et al., 2006) and smaller fibers (approximately 10–50 µm in diameter) *via* melt electrowriting (MEW) (Brown et al., 2016). Combined with biodegradable materials, such as polycaprolactone (PCL), a multi-scale scaffold architecture may provide an initial support structure while also providing a temporal degradation profile to stimulate enhanced long-term tissue development and growth (Agrawal and Ray, 2001).

Total disc replacements with regenerative constructs have been evaluated in both small animal models (Bowles et al., 2011; Gullbrand et al., 2018) and large animal models (Gullbrand et al., 2018; Yang et al., 2018). However, when treating spinal herniation, the symptomatic region of the IVD is frequently limited to a smaller annular defect (Knop-Jergas et al., 1996). An approach focused on localized regeneration of the AF defect may afford a less invasive solution to prevent re-herniation than whole disc replacement. However, this approach requires careful consideration of implant design and surgical attachment to maintain functional spinal biomechanics and implant loading that is conducive to tissue regeneration. To this end, animal models are invaluable evaluation platforms to translate novel orthopaedic treatments for human clinical applications. In particular, the ovine model for lumbar spine treatments is a widely accepted, well-established translational model that closely reflects the physiological scale and mechanical loading of the human spine (Wilke et al., 1997; Easley et al., 2008; Lindley et al., 2017; McGilvray et al., 2018). Accordingly, *ex vivo* ovine models can be leveraged to characterize the biomechanical effects of a repair strategy at the acute treatment phase. To evaluate temporal changes due to the healing response and high-cycle, physiological loading conditions, biomechanical characterization following *in vivo* implantation is also essential.

There remains an unresolved need to translate tissue engineered biomaterials into a surgically feasible strategy for IVD repair that can both elicit tissue regeneration and retain healthy spinal biomechanics. Accordingly, the objective of this study was to evaluate the relevant spinal biomechanics of a novel AF repair patch. An AF repair patch was designed and prescribed a previously-developed scaffold architecture to reproduce the pertinent mechanical properties of healthy AF tissue (Page et al., 2019). Comprehensive biomechanical characterization using an *ex vivo* ovine lumbar spine model was conducted to compare the biomechanical function of intact, injured, and repaired discs. Additionally, the biomechanics of a preliminary *in vivo* ovine lumbar spine study group was also characterized at 12 weeks post-implantation. A multi-scale scaffold architecture of 3DF and MEW fibers was used in the *in vivo* study to leverage the mechanical and biological functions of both fiber scales.

## 2 Materials and methods

The study design involved both *ex vivo* and *in vivo* evaluations of the novel IVD repair patch. The design and fabrication of the patch is described below, followed by the kinetic evaluation of the patch in a cadaveric ovine model. Given the promising results observed in these tests, a large animal cohort was used in a pilot study (n = 3) to determine the acute effects of implanting these patches *in vivo*. Post-sacrifice kinetic analyses were performed, followed by radiographic imaging, micro-computed tomography (micro-CT), histological imaging, and histomorphometric analyses (data provided in Supplemental Information to maintain brevity).

### 2.1 Annulus fibrosus repair implant

#### 2.1.1 Implant design

**FIGURE 1:**
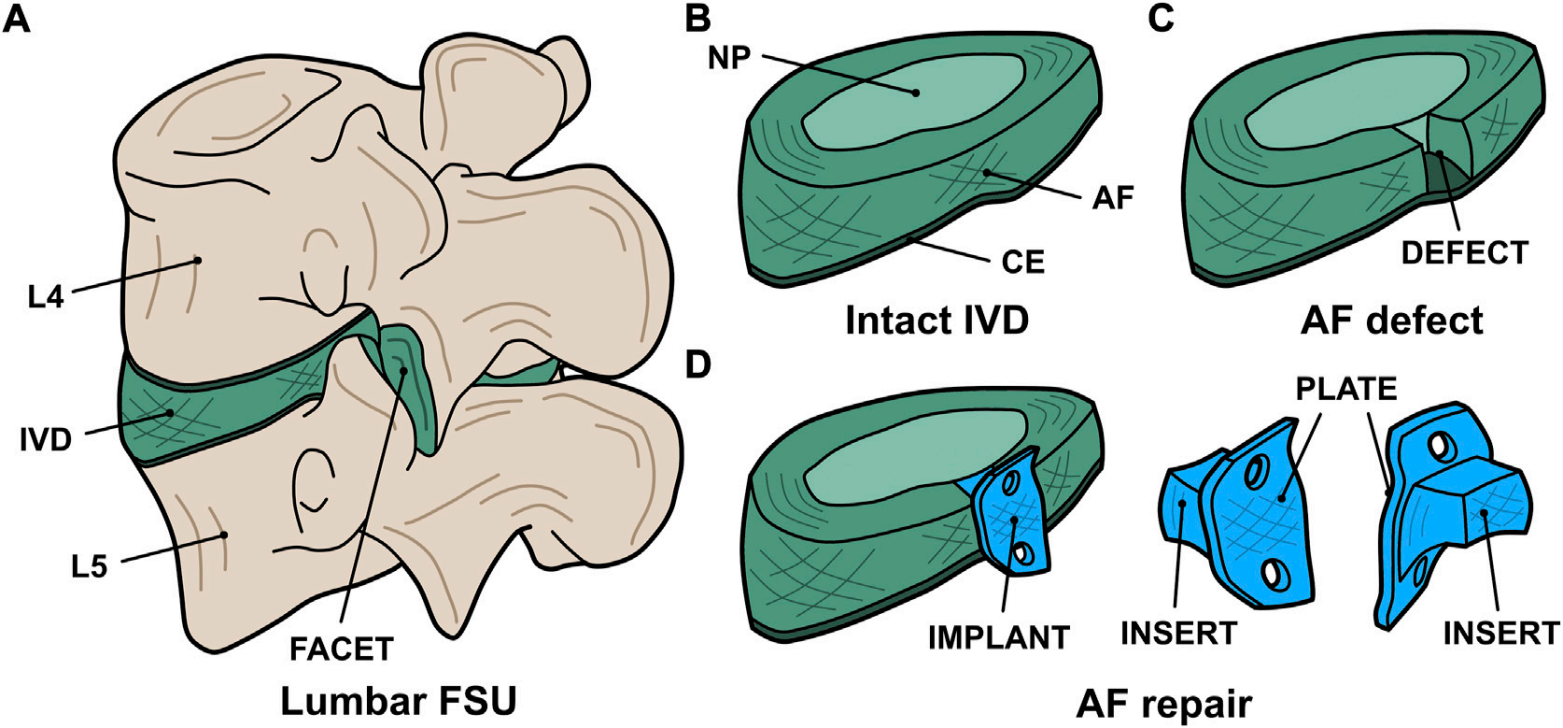
Digital renderings of the AF repair implant design: **(A)** the whole FSU indicating the lumbar levels (L_4_ and L_5_), intervertebral disc (IVD), and facet joint; **(B)** the intact IVD indicating the nucleus pulposus (NP), annulus fibrosus (AF), and cartilaginous endplate (CE); **(C)** the IVD with an annular defect in the posterolateral aspect; and **(D)** the treatment concept indicating the implant (blue) consisting of an insert to fill the defect and a plate to facilitate attachment to the vertebral bodies.

**FIGURE 2:**
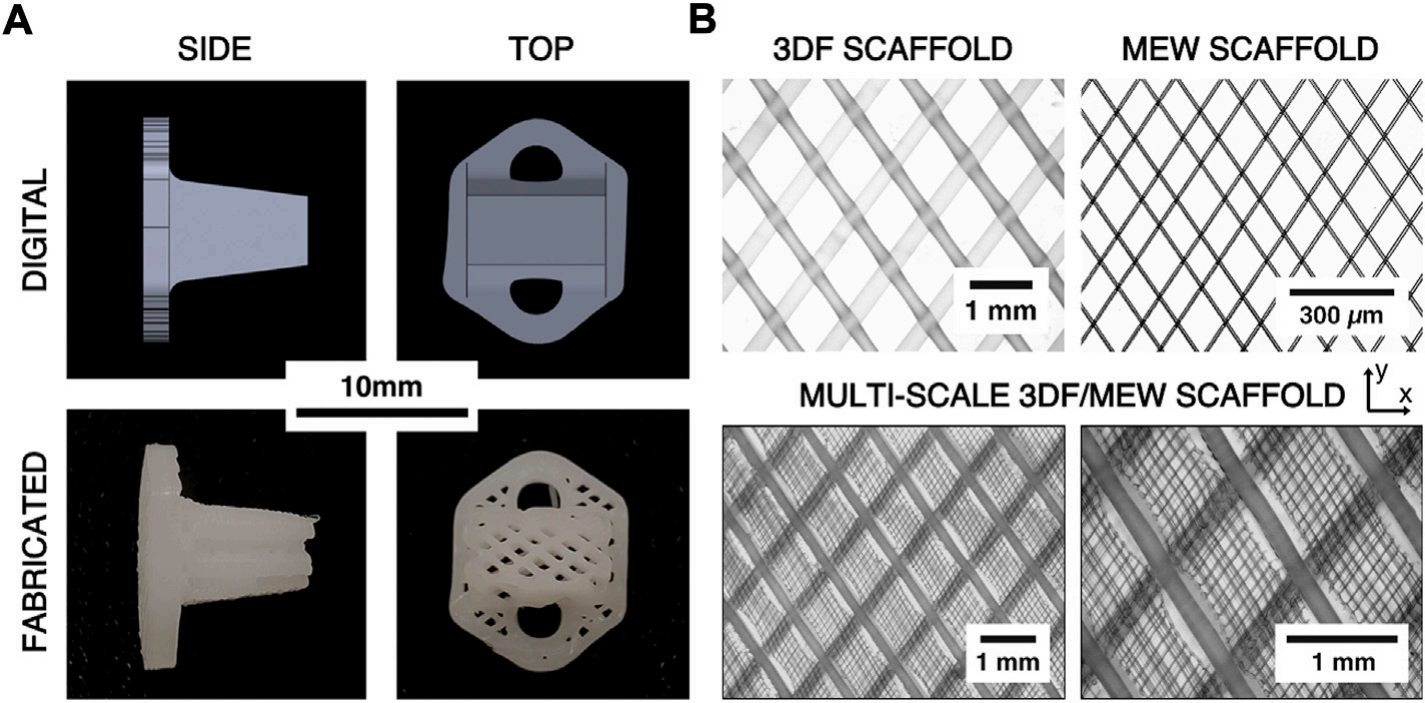
**(A)** Comparison of digital renderings of the ovine AF repair patch design and digital photographs of the printed scaffolds. **(B)** Example microscope images of 3DF, melt electrowriting (MEW), and hybrid 3DF/MEW scaffolds showing the architecture and relative scales of the 3DF and MEW components of the hybrid scaffold. The x- and y-directions represent the axial and circumferential directions on the AF repair implant, respectively.

A custom AF repair implant geometry was designed for human application with consultation from a board-certified, orthopaedic spine surgeon (VP). The resultant AF repair patch design comprised of an insert to fill the annular defect combined with an external plate to facilitate surgical attachment of the implant to the adjacent vertebral bodies with screws (Figure 1). Both the insert and plate were designed to be continuously fabricated with the same 3D printed architecture. This human implant design was translated to an ovine implant design (Figure 2A) with consultation from a board-certified, veterinary surgeon with specialization in large animal spinal surgery (JE). Virtual geometry of the implant was generated in Solidworks (2016 SP4.0, Dassault Systèmes, Vélizy-Villacoublay, France) based on a digital model of an ovine lumbar spine.

The ovine implant design was adapted for printability *via* three-dimensional fiber deposition (3DF) and prescribed a fibrous architecture that has previously been demonstrated to replicate the pertinent mechanical properties of native AF (Page et al., 2019). Specifically, the implant was composed of an angle-ply laminate, 3DF scaffold with lamellae in the axial-circumferential direction (fiber angle = ±34° from the circumferential direction, fiber spacing = 1.0 mm, layer height = 175 µm). The implant architecture was generated using a combination of BioCAD software (RegenHU, Villaz-Saint-Pierre, Switzerland) and a custom g-code algorithm (Python 2.7, Python Software Foundation, United States).

#### 2.1.2 Implant fabrication

Fabrication of 3DF implants was conducted using a 3DBiodiscovery bioprinter (HM-100 module, RegenHU Ltd., Villaz-Saint-Pierre, Switzerland; 27 gauge nozzle, nozzle length = 6.35 mm, extrusion temperature = 130°C, extrusion pressure = 100 kPa, translation rate = 3 mm/s, auger speed = 4.5 rev/min). All 3DF fabrication was conducted using polycaprolactone (average Mn 80,000, Sigma Aldrich, St. Louis, MO, United States). The substrate temperature was controlled (initial temperature of 20°C followed by a linear decrease of 0.33 C/min to a final temperature of 10°C) in order to achieve high quality fiber deposition throughout the printing process.

Multi-scale implants consisting of the same 3DF architecture as well as MEW fibers were fabricated for the *in vivo* study due to the biological relevance of MEW fibers during the healing response (Figure 2B). The hybrid implants were fabricated by first prefabricating sheets of MEW fibers in an angle-ply laminate architecture (fiber angle = ±34° from the y-direction, fiber spacing = 0.1 mm, number of bilayers = 20) using the MEW toolhead of a 3DBiodiscovery bioprinter (MESW module, RegenHU Ltd., Villaz-Saint-Pierre, Switzerland; 26 gauge nozzle, nozzle length = 15 mm, melt temperature = 65°C, extrusion pressure = 80 kPa, translation rate = 40 mm/s, voltage = 4.5 kV, collector distance = 3.0 mm). The MEW sheets were then manually inserted between each 3DF bilayer such that the two fiber architectures aligned (Figure 2B).

#### 2.1.3 Implant evaluation

To measure the resultant fiber diameters of the MEW and 3DF processes, ten (n = 10) additional samples of the MEW sheets and 3DF cruciform (without the MEW layers) were printed with the same prescribed architectural and process parameters as the implants. Only one bilayer of these additional samples were printed to improve image quality. Images of each sample were captured using a transmission light microscope (Olympus BH-2, Tokyo, Japan) and ten random fiber diameters were measured from each scaffold (excluding the initial layer of 3DF fibers) using ImageJ software (National Institutes of Health, Bethesda, MD, United States). The average fiber diameter and spacing for each type of scaffold were then calculated. To observe the hybrid scaffold architecture, three example scaffolds with the combined 3DF/MEW architecture were also fabricated and imaged using the same methods.

### 2.2 *Ex vivo* ovine lumbar spine model

#### 2.2.1 Sample preparation

**FIGURE 3:**
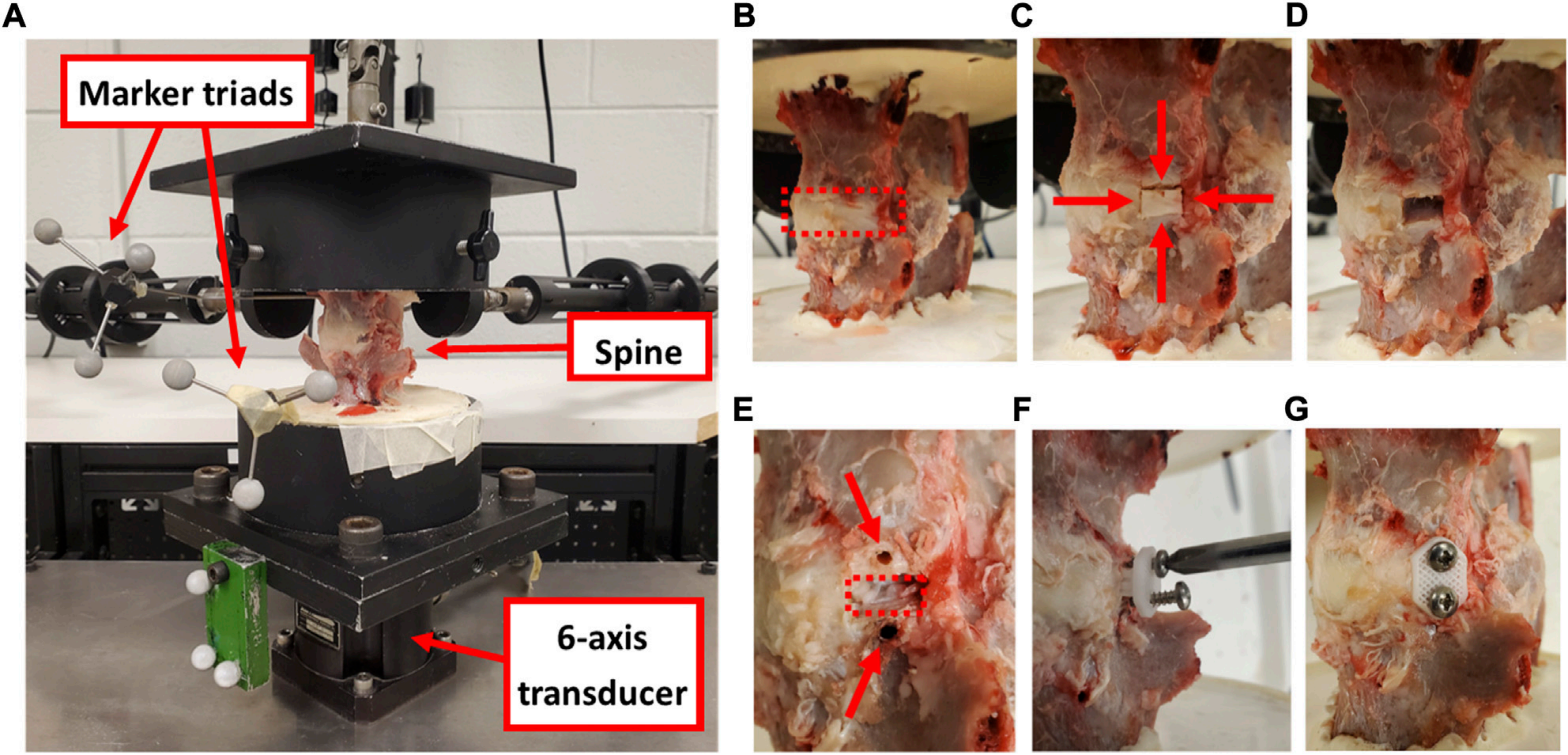
**(A)** Cadaveric ovine spine affixed to the testing frame with motion tracking marker triads and six-axis transducer that collected moment data during the assessments. **(B)** An intact FSU showing the disc space (red dashed box). **(C)** Two axial incisions in the AF were created 8 mm apart (shown with horizontal red arrows) and two circumferential incisions were created along the cartilage endplates (shown with vertical red arrows) to define the annular window. **(D)** The AF defect. **(E)** Two pilot holes were drilled for the screws (shown with red arrows) at the cranial and caudal aspects of the defect (shown with a red dashed box). **(F)** The implant was inserted into the defect and screwed in place. **(G)** The treated FSU with the annular repair patch fully implanted. Images c-h show the left lateral aspect of the disc.

Functional spine units (FSUs; n = 8) of the fourth and fifth lumbar levels (L4-L5 disc) were harvested *via* careful ex-plantation and fine dissection from eight skeletally mature ewes (3.5–4.5 years of age). Collection of FSUs was performed under approval from the Institutional Animal Care and Use Committee at Colorado State University (protocol #: KP1503). The spines were wrapped in saline-soaked gauze and stored frozen (−18°C) until biomechanical testing was performed. Each intact FSU was potted (Smooth-Cast^®^ 321, Smooth-On Inc., Macungie, PA, United States) at the cranial and caudal aspects for rigid mounting in a custom spine biomechanical testing system (Figure 3A) (McGilvray et al., 2018). Motion tracking markers were affixed to each vertebral body with Kirschner wires to track the motion of these FSU segments using a four-camera stereophotogrammetry system (Motion Analysis Corp, Santa Rosa, CA, United States).

#### 2.2.2 Biomechanical testing

Each FSU was tested in pure moment loading in three anatomical planes (Sakai, 2008): flexion and extension (Richardson et al., 2007), left and right lateral bending, and (O’Halloran and Pandit, 2007) left and right axial rotation. Specifically, five pre-conditioning cycles of loading to ±6.0 Nm (Oda et al., 1999; Kandziora et al., 2004; McGilvray et al., 2018) were applied across the FSU in each rotation plane and the reactionary moments were measured with a six degree of freedom load cell (1,000 lbf load capacity; Advanced Mechanical Technology, Inc. MC3-6-1K, Watertown, Massachusetts, United States). All other degrees of freedom remained free. For each biomechanical test, the recorded motion capture and moments of the fifth load cycle were processed using previously published methods to identify the range of motion (ROM), stiffness, and neutral zone (NZ) of the FSU (McGilvray et al., 2018). Briefly, the range of motion (ROM) was defined as the difference in angular position of the spine between −6 and +6 Nm of load. The limits of the neutral zone was defined as the central region of the moment-rotation curve bounded by inflections in the curve (identified as local maxima and minima of the second derivative of the moment-rotation curve). The neutral zone was defined as the angular rotation between these inflections and the NZ stiffness was defined as the least squares fit of a linear line to the NZ moment-rotation curve. The elastic zone (EZ) in the positive and negative directions (i.e., extension/left lateral bending/left axial rotation and flexion/right lateral bending/right axial rotation, respectively) were defined as the moment-rotation curve from +4.5 to +6 Nm and –4.5 to −6 Nm, respectively.

#### 2.2.3 Defect and treatment conditions

Following biomechanical testing of the intact FSU (Figure 3B), a section of IVD at the left lateral aspect (measuring 8 mm circumferentially, the full disc height axially, and the full AF depth radially such that the NP was exposed) was removed from the FSU using a custom guide (Figures 3C,D). The biomechanical evaluation protocol was repeated on the resultant partial discectomy model using the same testing protocol as the intact FSU. Pilot holes (1.9 mm diameter) were then drilled in the vertebral bodies and the AF repair implants were inserted within the discectomy and fixed with 2.88 diameter, 316 stainless steel screws (Figure 3E–G). A final series of biomechanical testing was conducted on the repaired FSU. Throughout biomechanical testing, sample hydration was maintained *via* physiologic saline spray at approximately 10 min intervals.

#### 2.2.4 Statistical analyses

Each group of biomechanical data was tested for normality using Anderson-Darling tests and Levene’s test was used to assess equal variance between groups for statistical comparison. An analysis of variance with repeated measures and Tukey *post hoc* comparisons were conducted between the means of the intact, defect, and treated groups for each biomechanical measure and loading condition. All statistical analyses were conducted with a significance level of *α* = 0.05.

### 2.3 *In vivo* ovine lumbar spine model

#### 2.3.1 Surgical approach

**FIGURE 4:**
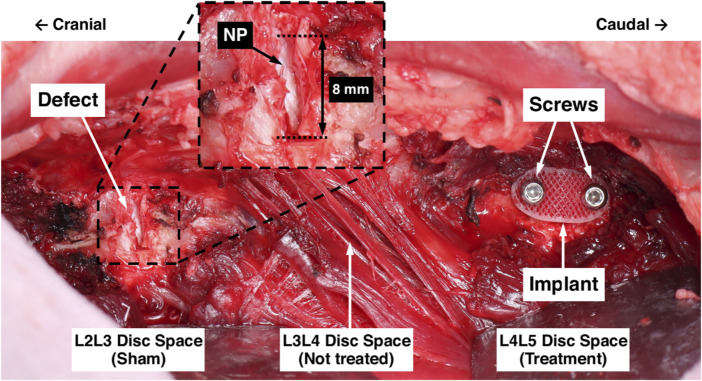
Surgical model for *in vivo* evaluation of the AF repair patch. The L_2_L_3_ disc space was prescribed a sham treatment and the defect is shown. The inset shows a magnified view of the defect, labelled are the 8 mm circumferential defect and the exposed NP (white tissue). The L_3_L_4_ disc space was not treated. The L_4_L _5_ disc space was prescribed the AF repair patch treatment following defect creation; the implant and screws are labelled.

Three skeletally mature ewes (3.5–4.5 years of age) were used for the pilot *in vivo* study under approval from the Institutional Animal Care and Use Committee at Colorado State University (protocol #: KP1262). Using a left lateral retroperitoneal approach, the L_2_ through L_5_ intervertebral spaces were exposed *via* a plane of dissection through the oblique abdominal muscles to the muscle plane ventral to the transverse processes. An annulotomy was performed at the left lateral aspects of the L_2_L_3_ and L_4_L_5_ disc spaces by excising an annular window (measuring 8 mm circumferentially and the full disc height axially) and removing the full radial thickness of the AF with pituitary rongeurs such that the NP was exposed. This defect was created to represent the surgical removal of a herniated section of disc. The L_2_L_3_ disc spaces were prescribed sham treatments, did not receive an AF repair patch, and remained empty (Figure 4). The L_1_L_2_ and L_3_L_4_ levels were not treated.

The L_4_L_5_ disc spaces were prescribed the AF repair patch treatment. Pilot holes were drilled in the adjacent vertebral bodies using a custom drill guide, an AF repair patch was inserted into the defect, and stainless steel cortical screws (2.7 mm diameter and 12 mm length) were inserted into the pilot holes to secure the implant, each attaining unicortical purchase. Prior to implantation, each implant was soaked for 30 min in 100% ethanol for sterilization. For preliminary comparisons of the pure 3DF and hybrid implant architectures, Animal 1 was prescribed a pure 3DF implant and Animals 2 and 3 were prescribed implants with the hybrid architecture.

#### 2.3.2 Sample preparation

Each animal was euthanized 12 weeks following surgery. This study group size (three sheep) and duration (12 weeks) served as a preliminary study to evaluate the surgical procedure and implant efficacy. Following euthanasia, the lumbar spine was harvested *en bloc* and finely dissected. The L_2_ to L_5_ vertebral bodies were axially transected to divide the whole lumbar spine (L_1_ to L_6_) into individual FSUs for evaluation.

#### 2.3.3 Sample evaluation

Non-destructive biomechanical testing of the FSUs was conducted using the same experimental protocol as the *in vivo* study (Section 2.2.2), except each FSU was only tested in the single, prescribed study condition (i.e. intact, defect, treatment). Following biomechanical testing, samples were processed for radiographic imaging, micro-computed tomography (micro-CT), histological imaging, and histomorphometric analyses. Detailed methods for these analyses are included as Supplemental Data.

#### 2.3.4 Statistical analyses

Due to the small sample size of the pilot study, the *in vivo* biomechanical data were not evaluated as statistical groups. However, statistical comparisons were made between each *in vivo* L_4_L_5_ FSU and the *ex vivo* groups (all L_4_L_5_ levels). Specifically, the biomechanical data for each *in vivo* treated level (L_4_L_5_) was individually compared to the intact, defect, and treated groups of the *ex vivo* study using one-sample Student’s t-tests (*α* = 0.05).

## 3 Results

### 3.1 Implant design and fabrication

The fabricated hybrid scaffolds clearly exhibited the intended 3DF scaffold architecture with no apparent gross defects and the MEW scaffold architecture was mostly retained within the 3DF layers (Figure 2). Digital microscope images revealed that some melting of the MEW sheets occurred near the succeeding 3DF fiber. Measurement of the 3DF samples yielded a mean fiber diameter of 230 µm (±20 µm standard deviation) and a mean fiber spacing of 990 µm (±10 µm standard deviation). The mean fiber diameter and fiber spacing measured from the MEW samples were 8.7 µm and 105 μm, respectively (±1.1 µm and ±22 µm standard deviations, respectively).

### 3.2 *Ex vivo* ovine lumbar spine model

**FIGURE 5:**
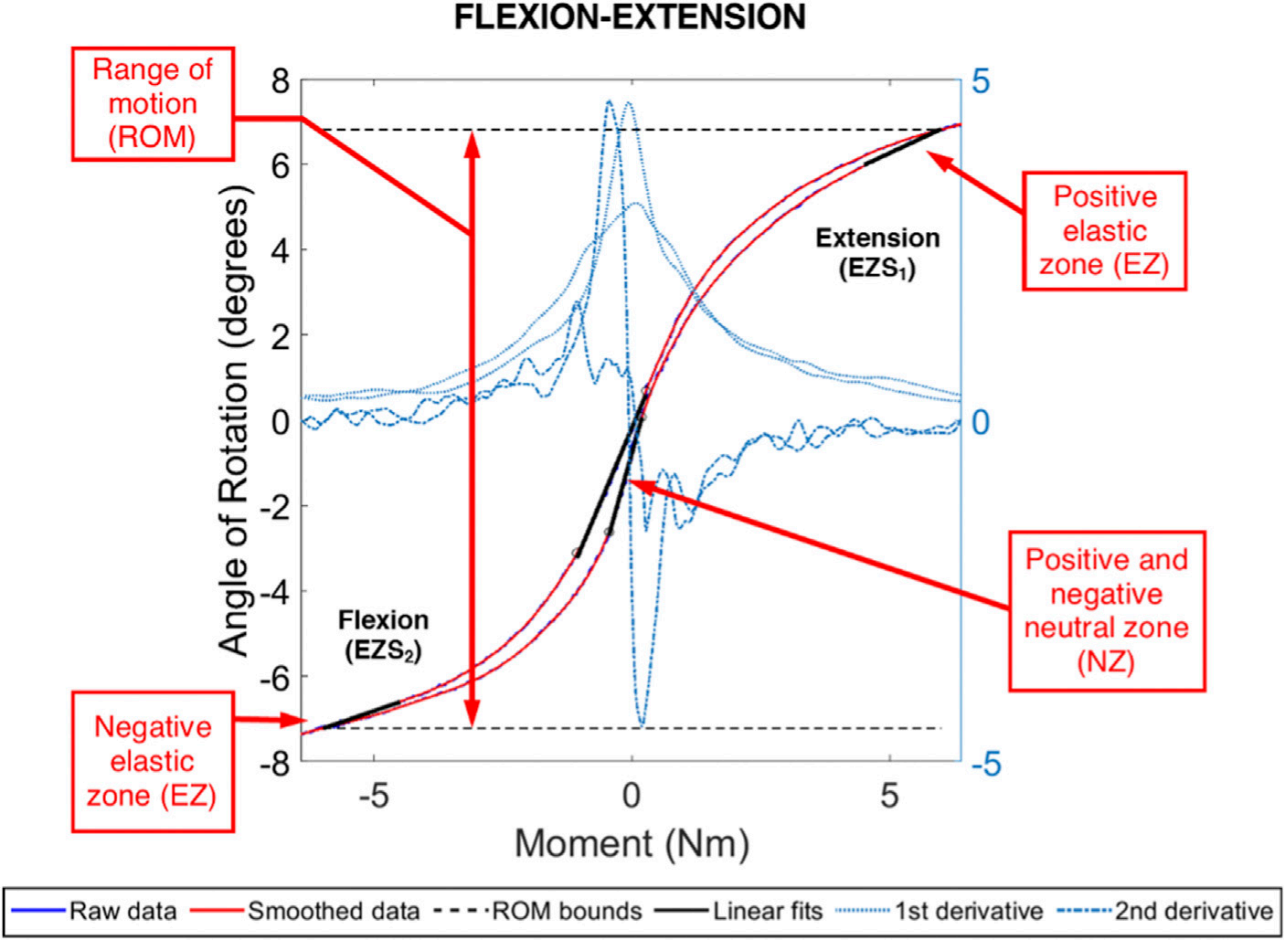
Example moment-rotation data for an ovine L4L5 FSU in pure, flexion-extension moment loading. Shown are the range of motion (ROM) and the positive and negative linear fits for the neutral zone (NZ) and elastic zone (EZ). The NZ size was evaluated between the local extrema in the central region and obtained using the second derivative of the smoothed data. The Ezs were evaluated between 4.5 Nm and 6.0 Nm (negative and positive).

An example moment-rotation diagram for one ovine L_4_-L_5_ FSU is shown in Figure 5. The measured FSU angle of rotation generally monotonically increased or decreased with moment loading, facilitating reliable calculation of the ROM, NZ size, NZ stiffness, and EZ stiffness. As compared to the flexion-extension and lateral bending data, a lower signal-to-noise ratio was observed in the axial rotation data. Summary data for the biomechanics of the three spine conditions are shown in Table 1.

**TABLE 1:** Kinetic measures of interest (mean ± standard deviation) for the healthy, defect, and treatment conditions of ovine L_4_L_5_ FSUs in flexion-extension (FE), lateral bending (LB), and axial rotation (AR).

FSU condition	Loading modality	ROM		NZ size		NZ stiffness [Nm/°]		EZS_1_ [Nm/°]		EZS_2_ [Nm/°]	
Healthy	FE	8.15	±2.30	2.53	±0.71	0.59	±0.27	4.18	±1.54	5.76	±1.44
	LB	9.57	±1.40	2.69	±0.82	0.59	±0.14	2.60	±0.41	2.86	±0.51
	AR	0.95	±0.27	0.28	±0.08	9.04	±3.39	16.28	±4.92	20.32	±8.99
Defect	FE	8.86	±2.29	2.45	±0.87	0.31	±0.14	5.15	±1.90	5.56	±1.54
	LB	12.32	±2.05	3.13	±0.40	0.25	±0.09	2.46	±0.52	3.29	±0.60
	AR	1.14	±0.29	0.27	±0.12	5.59	±2.08	13.07	±2.87	15.59	±5.73
Treatment	FE	8.62	±2.34	2.66	±0.86	0.50	±0.26	4.70	±2.71	4.81	±1.11
	LB	10.52	±1.93	2.78	±0.77	0.47	±0.18	2.37	±0.29	2.93	±0.50
	AR	1.12	±0.34	0.33	±0.13	6.15	±2.13	14.64	±8.09	13.92	±4.17

#### 3.2.1 Flexion-extension biomechanics

**FIGURE 6:**
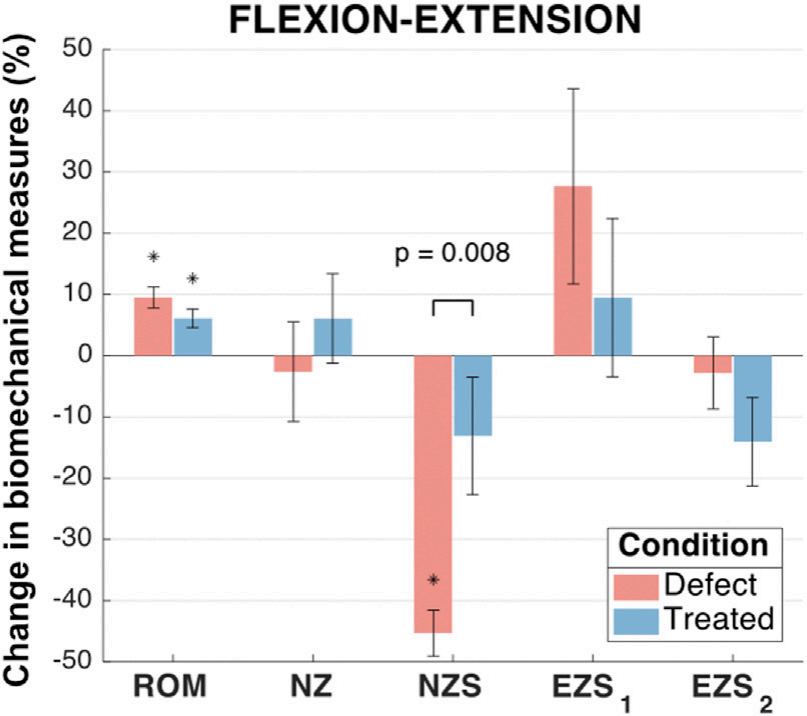
Flexion-extension biomechanical measures of interest for the defect and treated conditions relative to the intact condition. Error bars indicate the standard deviation for each group. Groups with a significant change in biomechanical measures from the intact case are indicated with asterisks (α = 0.05) and *p*-values are shown for significant differences between defect groups and treated groups (no *p*-value indicates no significant difference between groups).

In flexion-extension, ROM significantly increased for defect (*p* < 0.001) and treated (*p* = 0.003) groups in comparison to intact specimens (Figure 6). The mean increase in ROM of the treated group was less than the defect group (increases of 9.5% and 6.1%, respectively). However, the ROM of the treated group was not significantly different from the defect group (*p* = 0.12).

No significant differences were found between the mean flexion-extension NZ size of any groups (0.088 < *p* < 0.930; Figure 6). The NZ stiffness was significantly reduced in the defect group as compared to the intact group (mean NZ stiffness reduction of 45.3%; *p* < 0.001). However, the treated group (mean NZ stiffness reduction of 13.1%) was not significantly different from the intact group (*p* = 0.264) and was significantly different from the defect group (*p* = 0.008). The flexion extension elastic zone stiffnesses demonstrated no significant changes between any of the groups (0.08 < *p* < 0.88; Figure 6).

#### 3.2.2 Lateral bending biomechanics

**FIGURE 7:**
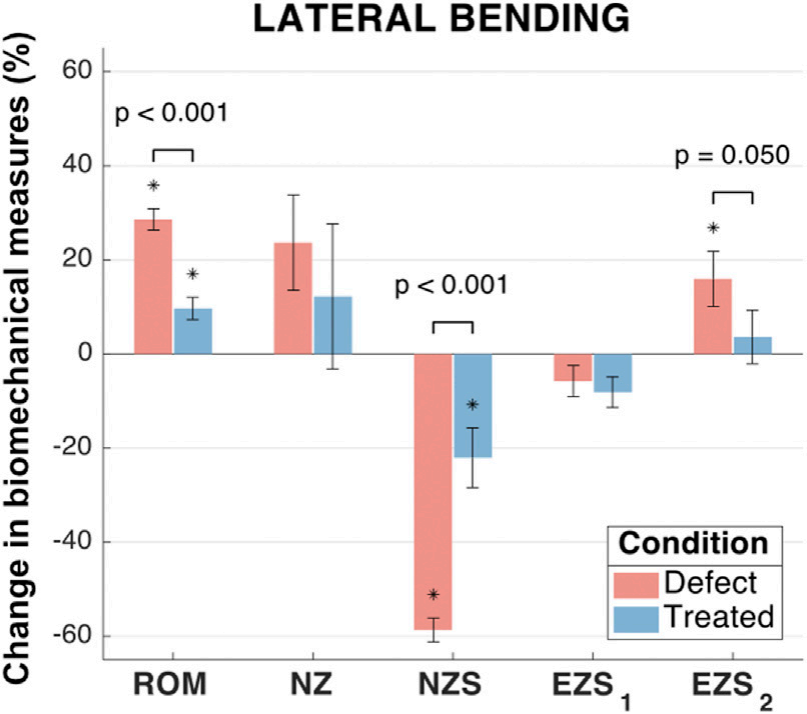
Lateral bending biomechanical measures of interest for the defect and treated conditions relative to the intact condition. Error bars indicate the standard deviation for each group. Groups with a significant change in biomechanical measures from the intact case are indicated with asterisks (α = 0.05) and *p*-values are shown for significant differences between defect groups and treated groups (no *p*-value indicates no significant difference between groups).

In lateral bending, significant differences were observed between the mean ROM of all three groups (Figure 7). As compared to the intact group, the mean ROM of the defect and treated groups significantly increased (by 28.6% and 9.7%, respectively; *p* < 0.001 and *p* = 0.006, respectively). Moreover, the mean change in ROM for the treated group significantly less than the mean change in ROM for the defect group (*p* < 0.001).

No significant differences were observed between the mean NZ size of any groups (Figure 7). However, the mean increase in NZ size of the defect group (23.7%) was greater than the treated group (12.2%). The NZ stiffness data yielded significant differences between all three groups (*p* < 0.001 and *p* = 0.004 for the defect and treated groups, respectively, as compared to the intact group). The mean reduction in NZ stiffness for the defect group (58.6%) was significantly greater than the treated group (22.1%; *p* < 0.001).

No significant differences were observed between any groups for right EZ stiffness and was associated with low magnitudes in the change of EZ stiffness (mean reductions in EZ stiffness of 5.8% and 8.1% for the defect and treated groups, respectively; *p* = 0.41 and *p* = 0.11, respectively; Figure 7). However, the mean left EZ stiffness for the defect group demonstrated a significant increase as compared to both the intact and treated groups (mean increase in EZ stiffness of 16.0% and 12.3%, respectively; *p* = 0.02 and *p* = 0.05, respectively).

#### 3.2.3 Axial rotation biomechanics

**FIGURE 8:**
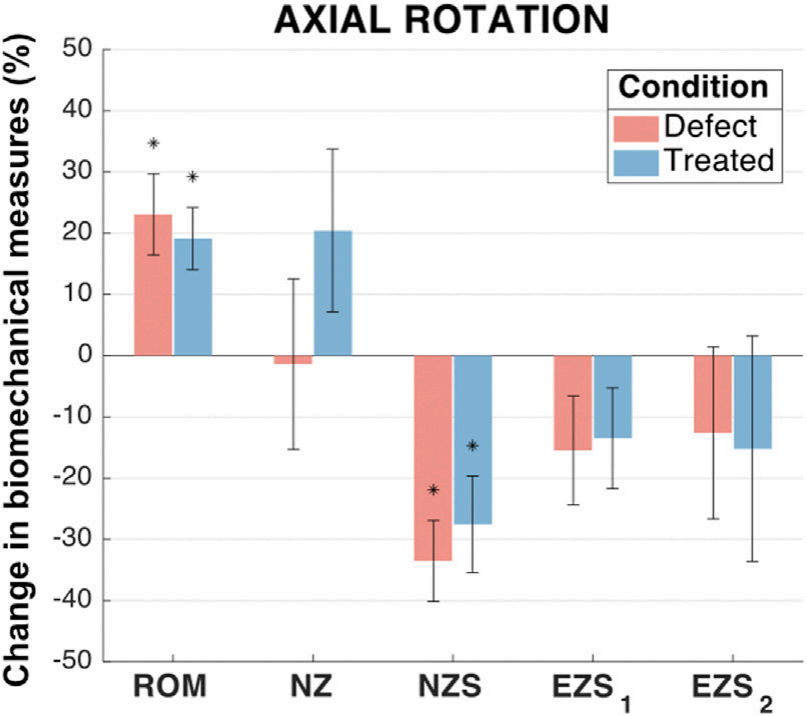
Axial rotation biomechanical measures of interest for the defect and treated conditions relative to the intact condition. Error bars indicate the standard deviation for each group. Groups with a significant change in biomechanical measures from the intact case are indicated with asterisks (α = 0.05) and *p*-values are shown for significant differences between defect groups and treated groups (no *p*-value indicates no significant difference between groups).

In axial rotation, no significant differences were observed between any groups for NZ size and both left and right EZ stiffness (Figure 8). The mean axial rotation ROM was significantly increased in the defect group (increase of 23.1%; *p* < 0.001) and treated group (increased of 19.1%; *p* = 0.001) as compared to the intact group. Similarly, when compared to the intact group, the defect and treated groups demonstrated significant decreases in the mean NZ stiffness (decreases of 33.5% and 27.5%, respectively; *p* < 0.001 and *p* = 0.001, respectively). In both ROM and NZ stiffness, the treated group mean was closer to the intact case than the defect group. However, there were no significant differences between the defect and treated groups.

### 3.3 *In vivo* ovine lumbar spine model

All three surgeries were performed without complication and the three sheep were ambulatory 2 h post-operative. No major complications were reported/observed during the 12 weeks in-life portion of the study. After sacrifice and fine dissection, fibrous tissue growth at the left lateral aspects of the sham and treated levels was visually observed in all three samples. The fibrous growth at the treated level consistently appeared larger than at the sham level. No abnormal excess fibrous tissue was observed at any of the intact or contralateral disc spaces. In the treated levels of Animals 1 and 3, the outer surface of the implant plate was visible within the fibrous growth. However, the implant was not visible in Animal 2.

#### 3.3.1 Flexion-extension biomechanics

In flexion extension, there were no clear trends in any of the biomechanical measures as a function of the FSU condition (i.e., intact, sham, or treated). Statistical comparison of the *in vivo* treatment levels (L_4_L_5_) to the *ex vivo* L_4_L_5_ groups (acute time point) demonstrated that the treatment ROM for all three animals was not significantly different to the intact spines (*p* ≥ 0.080 for all). For Animal 1 and Animal 3, the ROM was also not significantly different to the acute defect group (*p* = 0.346 and *p* = 0.194, respectively), but for Animal 2 the ROM was significantly different to the acute defect group (*p* = 0.022). The treatment NZ sizes were not significantly different to intact (*p* > 0.104 for all) or acute defect groups (*p* ≥ 0.250 for all) for all three animals. The treatment NZ stiffness for Animal 1 and Animal 2 was significantly different to the intact spine group (*p* = 0.006 and *p* = 0.010, respectively) and not significantly different to the acute defect group (*p* = 0.071 and *p* = 0.252, respectively). Conversely, the NZ stiffness for Animal 3 was not significantly different to intact spines (*p* = 0.208) and was significantly different to the acute defect group (*p* = 0.024). The EZ stiffnesses did not yield any clear trends. Notably, for Animal 3, all flexion-extension measures were not significantly different from the intact spine group (*p* ≥ 0.104 for all).

#### 3.3.2 Lateral bending biomechanics

In lateral bending, all five biomechanical measures appeared to follow similar trends between conditions as the *ex vivo* data. Specifically, the treated levels generally caused a partial correction or over-correction of the changes in ROM, NZ size, and NZ stiffness induced by the defect. As compared to the corresponding *ex vivo* groups, the intact ROM, NZ size, and EZ stiffness appeared to have more variance in the *in vivo* data. Statistical comparison of the *in vivo* treatment levels (L_4_L_5_) to the *ex vivo* groups (acute time point) demonstrated that all three animals had treatment ROM significantly different to the acute defect group (*p* ≤ 0.004 for all). Animals 1 and 3 had treatment ROM not significantly different to the intact spine group (*p* = 0.537 and *p* = 0.486, respectively), but Animal 2 had treatment ROM significantly different to intact (*p* < 0.001). The treatment NZ size for all three animals was significantly different to the acute defect group (*p* < 0.001 for all). However, the treatment NZ size was significantly different to the intact group for Animal 2 and Animal 3 (*p* = 0.002 and *p* = 0.007, respectively) and not significantly different to intact for Animal 1 (*p* = 0.121). No clear trends were apparent for the NZ size or EZ stiffnesses in lateral bending. All five biomechanical measures for Animal 2 were significantly different to the acute defect group (*p* ≤ 0.022 for all), but statistical differences varied as compared to intact spines.

#### 3.3.3 Axial rotation biomechanics

In axial rotation, the *in vivo* ROM, NZ stiffness, and EZ stiffnesses appeared to follow the same trend between conditions as the *ex vivo* data. In particular, the ROM appeared to be distinctly larger for the defect spines as compared to the intact spines and the treated spines had similar ROM to the intact spines. Additionally, the intact and defect ROM appeared to have more variance in the *in vivo* as compared to the corresponding *ex vivo* groups. Statistical comparison of the *in vivo* treatment levels (L_4_L_5_) to the *ex vivo* groups (acute time point) demonstrated that all three animals had treatment ROM significantly different to the intact spine group (*p* ≤ 0.026 for all). Further, Animal 1 and Animal 2 had treatment ROM not significantly different the acute defect group (*p* = 0.084 and *p* = 0.513, respectively). The treatment ROM for Animal 3 was significantly different to the acute defect group (*p* < 0.001). The treatment NZ size was not significantly different to either the intact or acute defect groups for Animal 1 (*p* = 0.207 and *p* = 0.491, respectively) and Animal 2 (*p* = 0.365 and *p* = 0.409, respectively). Conversely, Animal 3 had treatment NZ size significantly different to intact and acute defect groups (*p* = 0.001 and *p* = 0.006, respectively). The treatment NZ stiffness and EZ stiffnesses were significantly different to the intact spine group for all three animals (*p* ≤ 0.038 for all). The treatment NZ stiffness and EZ stiffnesses were also significantly different to the acute defect group for Animal 1 (*p* ≤ 0.030 for all) and Animal 3 (*p* ≤ 0.008 for all) but were not significantly different to the acute defect group for Animal 2 (*p* ≥ 0.14 for all). For Animal 3, all five biomechanical measures were significantly different to both the intact (*p* ≤ 0.007 for all) and acute defect groups (*p* ≤ 0.008 for all). For Animal 2, all five biomechanical measures were not significantly different to the acute defect group (*p* ≥ 0.140 for all).

#### 3.3.4 Other post-sacrifice analyses

**FIGURE 9:**
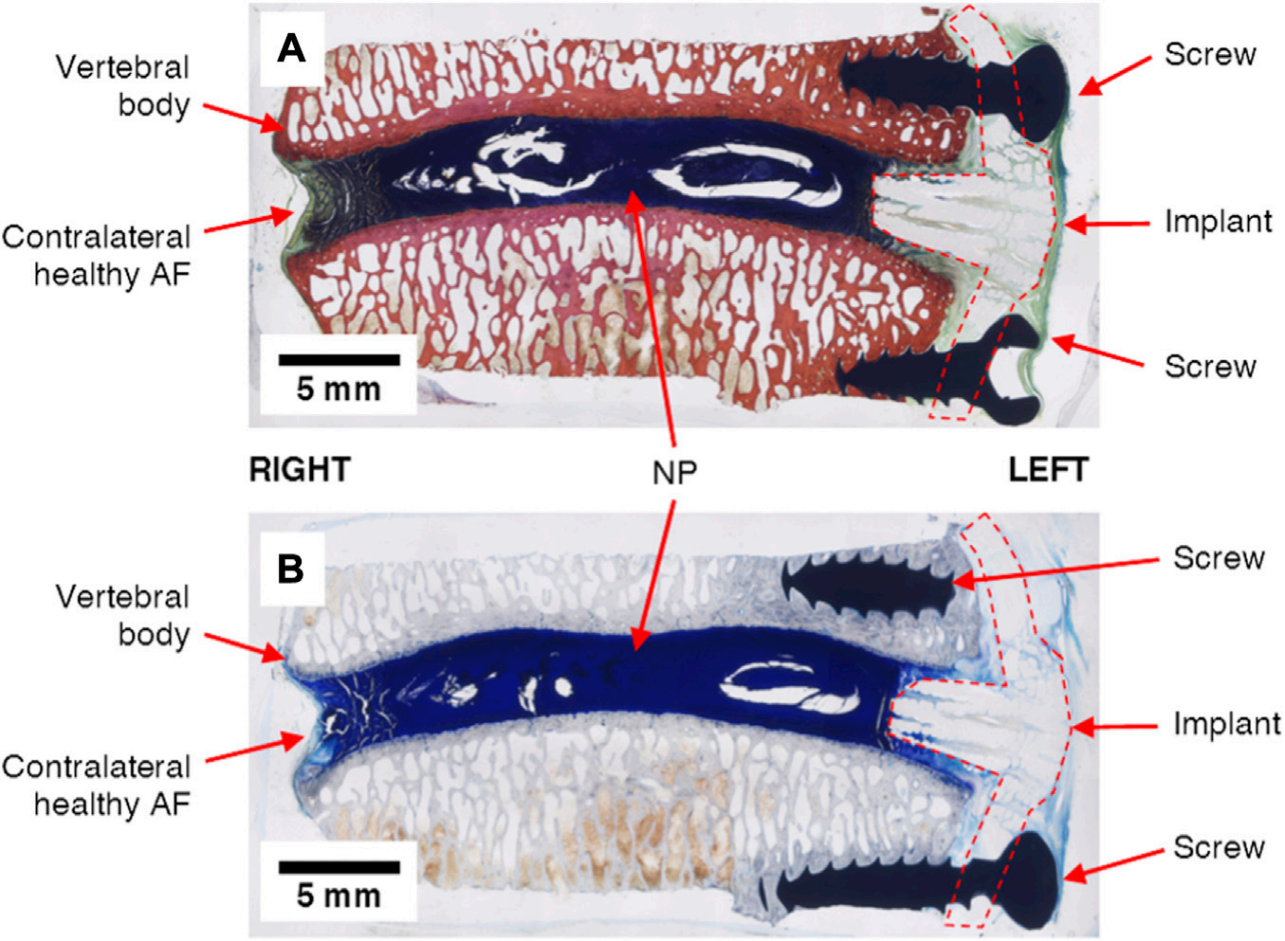
Example undecalcified histological images of treated IVD levels (L_4_L_5_, Animal 3) strained with **(A)** Sanderson’s Rapid Bone Stain and **(B)** Toluidine Blue Stain. The nucleus pulposus (NP), annulus fibrosus (AF), metal screws and repair patch (“implant”) are clearly visible in each image.

Detailed methods and results for the post-sacrifice radiographic imaging, micro-computed tomography (micro-CT), histological imaging, and histomorphometric analyses are included as Supplemental Data, and a short summary of these findings is given here. The measured disc heights from the 0-week time point radiographs exhibited no statistically significant differences compared to the 12-week time points for the sham conditions (0.16 < *p* < 0.64), intact conditions (0.12 < *p* < 0.77) and the treated conditions (0.43 < *p* < 0.80). Micro-computed tomography revealed dense tissue masses near the implant site and outside of the disc space in the treated levels of all three animals. Conversely, no dense tissues were observed in any of the other lumbar levels. These observations were consistent with histological analyses. Animals 1 and 3 exhibited masses at the treatment site generally consisting of soft tissues and Animal 2 demonstrated notable calcified tissue formation near the treatment site. Histological imaging also revealed that the implant plate was retained by the screws in Animals 2 and 3, however, screw loosening was observed in Animal 1. Example histological images that demonstrate a treated IVD are shown in Figure 9.

## 4 Discussion

In this study, an implant for repair of the annulus fibrosus (AF) was designed and fabricated using a novel tissue engineering scaffold architecture, tested, and translated to an ovine model. A surgical approach for the AF repair strategy was developed using cadaveric ovine lumbar spines and the *ex vivo* biomechanics of intact, injured, and treated lumbar spines (L4L5) were evaluated. A preliminary *in vivo* ovine lumbar spine model study of the AF repair strategy was conducted and biomechanical testing was performed 12 weeks post-implantation.

### 4.1 Implant design and fabrication

Overall, the fabricated implants effectively reproduced the designed 3DF architecture. This scaffold architecture used for the implants was identified in previous work to reproduce the pertinent mechanical properties of the AF (Page et al., 2019). However, the measured 3DF fiber diameters were smaller than what has been previously reported. Computational work on the biaxial mechanics of angle-ply laminate scaffolds has shown that a smaller fiber diameter reduces the overall scaffold compliance and increases the asymmetry of the biaxial stiffnesses (i.e. axial-to-circumferential biaxial stiffness ratio) (Page and Puttlitz, 2019). Further, computational modelling has predicted that increased implant compliance may be beneficial for delivering mechanoregulatory stimulus to resident cells (Page et al., 1152). Microscopic imaging of samples of the multi-scale scaffolds demonstrated that the MEW fibers were retained within the scaffold (Figure 2). Thus, this approach allows the development of a biomimetic scaffold that can span numerous length scales to engineer the cellular mechanical environment, a capability that can be leveraged to manipulate the resident (or implanted) cells to create localized tissue architectures that approximate the healthy, native tissue.

### 4.2 *Ex vivo* ovine lumbar spine model

The intervertebral kinetic data for the intact ovine L4L5 FSUs was consistent with previously reported values (Wilke et al., 1997; Easley et al., 2008). Overall, the *ex vivo* biomechanics demonstrated that the AF repair implant generally maintained the intact biomechanics of the FSU. Notably, in every instance in the study where the defect group had significantly different biomechanics to the intact spines, the treated group more closely approximated the intact biomechanics as compared to the corresponding defect group. For example, although the mean lateral bending ROM of the treated group was significantly different to the intact spines (9.7% increase in ROM as compared to intact), the scaffold’s effect on the spinal kinetics represented a substantial improvement as compared to the mean of the defect group (28.6% increase in ROM as compared to intact).

The kinetic evaluations indicate that all three loading modalities were important considerations for assessing the biomechanical influence of the defect and treatment. The results suggest that the NZ stiffness and ROM may be the most pertinent of the considered biomechanical measures for AF repair. In particular, lateral bending appeared to demonstrate the greatest biomechanical changes due to the defect and implant. This can be explained by the lateral location of the defect; the distance to the neutral axis of bending (and therefore the influence on the areal moment of inertia) is maximized in lateral bending. Across all three loading modes, the ROM and NZ stiffness were the biomechanical measures most influenced by the defect and treatment; the defect group generated mean changes to the ROM and NZ stiffness that were significantly different to the intact group in all three loading modes. From a mechanistic perspective, the changes in ROM and NZ stiffness could be explained by the discontinuity of the structural fibers that was induced by the defect, an effect that was not recovered by the treatment. Recruitment of these structural fibers (e.g., collagen) has be demonstrated to be a critical factor for FSU biomechanics (Ayturk et al., 2010). Moreover, the AF implant was designed to replicate the elastic zone mechanical properties of the AF. Therefore, the implant may not be able to reconcile the neutral zone behavior of the AF, which is dictated by the toe-region of the nonlinear AF elasticity profile.

### 4.3 *In vivo* ovine lumbar spine model

**FIGURE 10:**
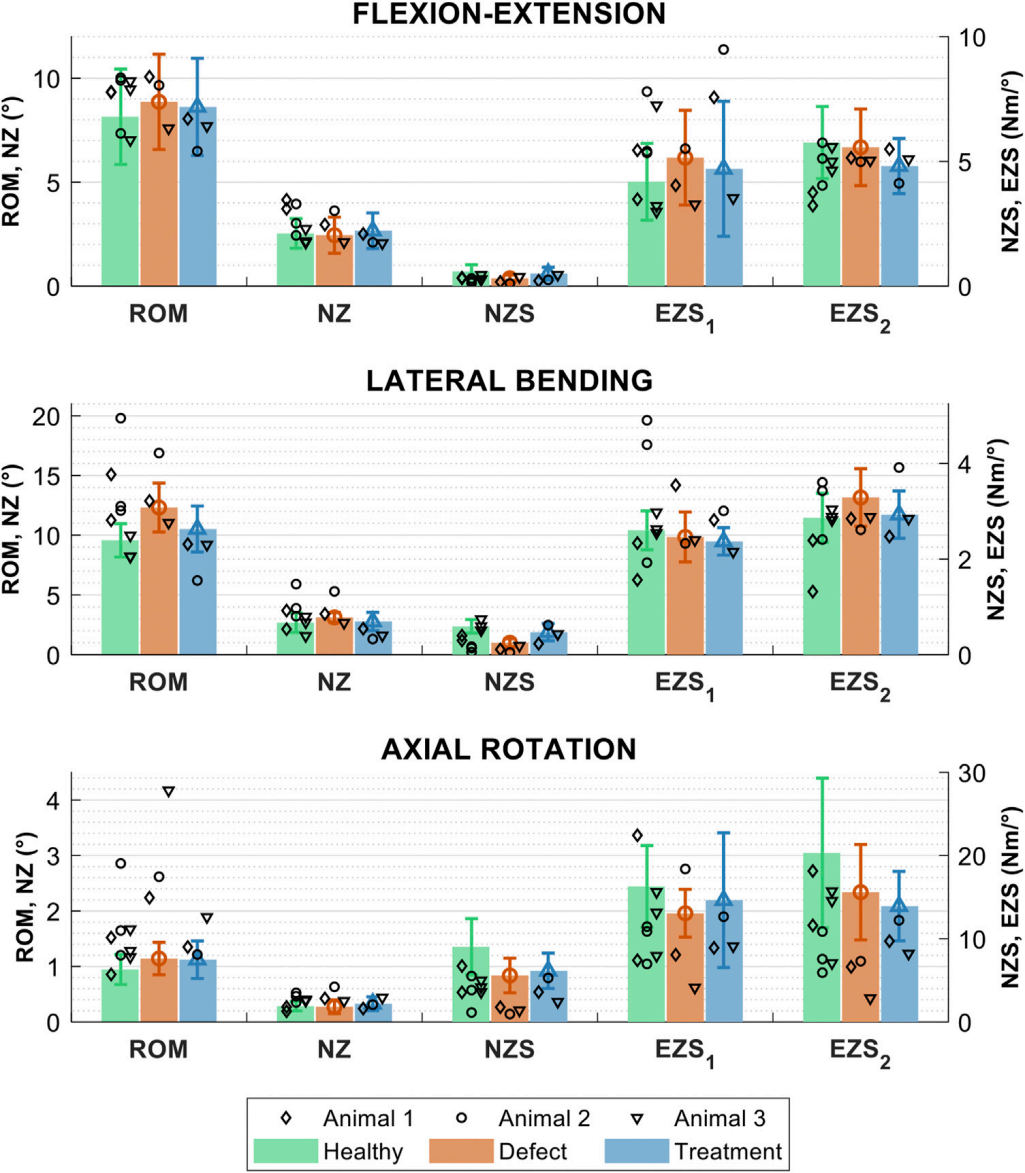
Biomechanical measures of interest for the healthy, sham, and treatment lumbar FSUs conditions in three loading modes: flexion-extension, lateral bending, and axial rotation. The biomechanical measures are the range of motion (ROM), neutral zone (NZ) size, NZ stiffness, and the positive and negative elastic zone stiffnesses (EZS_1_ and EZS_2_). Data are shown for the three *in vivo* studies (study 1 = ◊, study 2 = ○, and study 3 = ▿) and *ex vivo* study groups (group means are shown with solid bars). Error bars indicate the standard deviation for the *ex vivo* groups.

In general, the biomechanical measures (ROM, NZ size, NZ stiffness, and EZ stiffnesses) of the FSUs from the *in vivo* study (L_1_L_2_ to L_5_L_6_ discs) were similar in magnitude to the intact, defect, and treated FSUs in the *ex vivo* study groups (Figure 10). The greatest changes in biomechanics due to the defect appeared to occur in axial rotation and the ROM and NZ stiffness measures for all loading modalities. The biomechanical data from the *in vivo* study exhibited a notably larger variation as compared to the *ex vivo* study, which can be primarily attributed to (Sakai, 2008): temporal changes during the *in vivo* study (Richardson et al., 2007); greater inconsistency of the surgical approach in the *in vivo* setting as compared the cadaveric (*ex vivo*) setting; and (O’Halloran and Pandit, 2007) use of different lumbar levels in the *in vivo* study. However, a larger study group with greater statistical power would be necessary to explicate the validity of the trends in the preliminary *in vivo* data.

Radiographic measurements demonstrated no clear changes in disc height or morphology of any disc level between the 0-week and 12-week time points (see Supplemental Data), a finding that indicated that the implant was able to support the dynamic, *in vivo* loads that were place upon it. . The acute (12 weeks) nature of this pilot study most likely precluded substantial advancement of the degenerative sequela (especially in the defect case) and a longer-term study should be performed to evaluate the implant’s ability to arrest the degeneration cascade.

Detachment of the implant from the screws was observed in Animal 1, and Animal 2 exhibited calcified tissue masses at the treatment site as well as the intact and defect levels (see Supplemental Data). These observations are consistent with numerous biomechanical measurements that deviated from the *ex vivo* study group as well as the other *in vivo* study animals. However, it is unclear if the observed pathologies in Animal 2 were present prior to surgery or were a result of the treatment and/or sham. Again, a larger, sufficiently powered large animal cohort should be performed to further elucidate some of these mechanisms and provide feedback for future design and implantation strategy iterations.

### 4.4 Limitations

The primary limitation of this study to investigate the repair of AF herniation was the translatability of the large animal model to human application. Due to limited surgical access of the posterior disc in the ovine model, the implant was placed at the left lateral aspect of the IVD. However, disc herniation most frequently occurs near the posterolateral aspect of the IVD in humans (Knop-Jergas et al., 1996). The variation in implant location may have associated biomechanical effects (such as higher lateral bending loads in the ovine model vs higher flexion-extension bending loads in the human location placement) and a laminectomy may also be required to reach the surgical site. Additionally, to translate the repair strategy to clinical application, specific patient selection criteria need to be developed for the treatment, such as the nature, extent, and location of the AF defect.

The *in vivo* model was able to characterize the spinal biomechanics at a prescribed time point after the surgery, however, was unable to directly compare intact, injured, and treated conditions of each specific FSU. In this preliminary *in vivo* study, only three animals were investigated, and equivalent comparisons could not be drawn across different lumbar levels or between different animals. Resultantly, only basic comparisons of the biomechanical measures could be made. As stated above, a larger *in vivo* study group will be required to provide sufficient statistical power in order to provide a rigorous evaluation of the AF repair patch. The lack of randomization of FSU conditions in the *ex vivo* study may have also been a confounding factor in this study and should be addressed in a larger study group.

The results of this study suggest that modifications to the repair strategy are necessary to address the observed extrusion of the implant from the disc space. For example, the durability of the implant may be enhanced and/or the surgical attachment approach may be revised and evaluated with cyclic *ex vivo* testing. Further, more advanced animal studies may be undertaken by implementing the repair strategy on degenerative spines to assess the IVD patch in a more clinically-relevant model. A degenerative model could also be coupled with a NP repressurization strategy to afford a more holistic scheme for repair of IVD hernia.

## 5 Conclusion

Overall, this study demonstrated the design, fabrication, *ex vivo* evaluation, and implementation of a novel AF repair implant in a large animal model. A surgical implantation technique was developed using an ovine lumbar spine model and subsequent biomechanical testing demonstrated functional efficacy of the implant. Implementation of the repair strategy in a large animal model allows for iterative improvement to the implant design and a platform for clinical translation of a novel AF repair scaffold strategy. Ultimately, the developed approach to AF repair holds the potential to alleviate pain in severe cases of IVD herniation while maintaining the long-term biomechanical function of the spine and preventing symptomatic re-herniation.

## Data Availability

The original contributions presented in the study are included in the article/Supplemental Material, further inquiries can be directed to the corresponding author.
