# Supplementary material for: Biomechanical evaluation of a novel repair strategy for intervertebral disc herniation in an ovine lumbar spine model
Source: Front Bioeng Biotechnol. 2022 Oct 25;10:1018257. doi: 10.3389/fbioe.2022.1018257 (PMC9641051; doi:10.3389/fbioe.2022.1018257)
Supplement: Supplementary file 1 [file Table1.DOCX]

Supplementary Material

# Supplementary materials and methods

## *In vivo* ovine lumbar spine model

### *Radiographic imaging*

Each Animal underwent ventrodorsal and lateral radiographs every two weeks during the *in vivo* study. Immediately following euthanasia, whole spines were radiographed in the sagittal and coronal planes. Disc heights were measured from the radiographic images at the 0-week (i.e., post-operative) and 12-week (i.e., post sacrifice) time points. Specifically, the disc height was calculated as the mean distance between the vertebral endplates at five equally spaced locations in the disc in the sagittal plane with ImageJ software (National Institutes of Health, Bethesda, MD, USA). Student’s t-tests were used to compare the disc heights at the 0-week and 12-week time points.

### *Micro-computed tomography*

Following biomechanical evaluation, the whole disc was dissected from each FSU by transecting the vertebral bodies in the transverse plane approximately 5mm from the disc space (Supplementary Figure 1). These samples were placed in 10% neutral-buffered formalin (10% NBF) for two weeks. During fixation, each level was evaluated using micro-computed tomography (micro-CT) to detect any adverse bone formation in the disc space. The metal screws used to surgically attach the implant were left in place during micro-CT scanning to ensure that the implant remained in place and provided assurance that the interfaces between the implant and adjacent tissues would remain intact for subsequent histological imaging. The micro-CT scans were conducted with 37 x 37 x 37 μm voxel size, 70 kV potential, 500 ms integration time, 114 μA intensity, and 7.98 W power. For each level, the bone volume was quantified in two regions of interest (ROIs), defined as the left and right aspects of the whole disc space. The bone volume within each ROI was computed as the volume with a mineral density between 220 and 1000 mg/cm3 hydroxyapatite. Following micro-CT imaging, each FSU section was dissected down to the location of the implant, defect, or corresponding disc tissue (Supplementary Figure 1) and returned to the 10% NBF solution.

### *Histology*

For histological evaluation, the samples were dehydrated in graded ethanol and cleared with Histoclear (National Diagnostics, Atlanta, GA, USA) followed by infiltration and embedding in methyl methacrylate (MMA; Acrylosin Hard, Dorn and Hart, Loxley, Alabama, USA). At least two sections were cut through the center of each disc ROI in the coronal plane. Initial sections of approximately 300 µm were obtained using a diamond blade saw (Exakt Technologies, Oklahoma City, OK), ground, and polished to a final thickness of 60 - 70 µm using a microgrinder (Exakt Technologies, Oklahoma City, OK). One half of the sections for each sample were stained with Sanderson's rapid bone stain (SRBS; Dorn and Hart Microedge Inc., Villa Park, IL, USA) and counterstained using Van Gieson’s stain (VGCS; Dorn and Hart Microedge Inc., Villa Park, IL, USA) to differentiate cells, tissue structure, cartilage, collagen, and bone. The other sections for each sample were stained with toluidine blue (TBS; Sigma Aldrich, St. Louis, MO, USA) to detect proteoglycan content.

### *Histomorphometry*

Histomorphometric measurements were made using Image Pro software (Media Cybernetics, Silver Spring, Maryland, United States) to quantify the tissue structure within each disc level of each study. In the histological section plane, the ROIs for histomorphometric analyses was defined as: (1) the full area of the left AF; (2) the full area of the right AF; and (3) the full area of the NP. An additional ROI was defined for the Sanderson’s Rapid Bone Stain (SRBS) sections of the treated levels to characterize the implant, screws and tissue observed outside of the left lateral aspect of the IVD; this ROI was bounded by the cranial edge of the cranial screw, the caudal edge of the caudal screw, the vertebral body, and the peripheral surface of the implant/tissue mass. Within each ROI stained with SRBS, the histomorphometric parameters measured were: (1) percent proteoglycan-rich soft tissue area (as defined by blue stain), (2) percent fibrous soft tissue area (as defined by green stain), (3) percent bone area (as defined by red stain), (4) percent scaffold area, and (5) percent screw area. The histomorphometric parameters for ROIs in the Toluidine Blue Stain (TBS) sections were: (1) percent of proteoglycan-rich soft tissue (as defined by blue stain) and (2) percent scaffold area.

# Supplementary results

## *In vivo* ovine lumbar spine model

### *Radiographic imaging*

Radiographic images exhibited no clearly identifiable, deleterious effects in any of the disc spaces at any time point in the study. As compared to the healthy and sham levels, a slightly greater radiopacity was observed in the regions surrounding the treated levels at the 12-week time point in two of the three Animals (Supplementary Figure 2; Supplementary Table 1). These regions were not identifiable in the 0-week time point radiographs. The height of the L_1_L_2_ discs (healthy) in Animals 2 and 3 could not be reliably measured from the 0-week time point radiographs. The measured disc heights from the 0-week time point radiographs exhibited no statistically significant differences compared to the twelve-week time points for the sham conditions (0.16 < p < 0.64 for the L_2_L_3_ levels), healthy conditions (0.12 < p < 0.77 for the L_3_L_4_ levels) and the treated conditions (0.43 < p < 0.80 for the L_4_L_5_ levels).

### *Micro-computed tomography*

Bone volumes measured by the micro-CT analyses are shown in Supplementary Table 2. All discs in Animal 1 exhibited a bone volume less than or equal to 0.01%. In Animal 2, the bone volume was less than or equal to 0.01% for the L_4_L_5_ disc (treated), L_3_L_4_ disc (healthy), and the right side of the L_2_L_3_ disc (sham level, contralateral side). The left side of the L_2_L_3_ disc (sham level, defect side) exhibited a marginally increased bone volume (0.05%). The L_1_L_2_ and L_5_L_6_ discs (both healthy) had the greatest measured bone volumes (range 0.07% to 0.95%). In study Animal 3, the bone volume of the L_2_L_3_ disc, L_3_L_4_ disc, and the right side of the L_1_L_2_ disc was less than or equal to 0.01%. The remaining healthy discs (left side of the L_1_L_3_ disc and the L_5_L_6_ disc) had measured bone volumes ranging from 0.03% to 0.43%. The L_4_L_5_ disc (treated) had measured bone volumes of 0.11% and 0.04% at the left side (defect) and right side (contralateral), respectively. In all three Animals, artefacts in the micro-CT scans associated with the metal screws were observed for the L_4_L_5_ discs. In study Animals 1 and 3, the total measured volume of L_4_L_5_ discs (1253 mm^3^ and 1165 mm^3^) were within the range of the other measured discs (ranges of 882 to 1673 mm^3^ and 892 to 1674 mm^3^). However, in study Animal 2, the total volume of the L_4_L_5_ disc (916 mm^3^) represented the minimum value for all measured discs (range 1033 to 1266 mm^3^). Dense tissue masses were observed near the implant site and outside of the disc space in the treated levels of all three Animals (Supplementary Figure 2). These dense regions were largest in transverse plane cross-section near the screw sites. No similar dense tissues were observed in any of the other lumbar levels.

### *Histology*

In the histological sections with SRBS, calcified tissue was clearly identified with red stain and soft tissues were identified with green and blue stain (Supplementary Figure 4). Soft tissues were also identified in blue with the TBS, particularly in the nucleus region of the disc and reducing in intensity radially from the inner to outer AF in healthy sections (Supplementary Figure 5). Similarly, the healthy AF demonstrated a radial gradient from blue color to green with SRBS. Healthy AF consistently demonstrated a lamellar structure in all histological sections (Supplementary Figure 4 and Supplementary Figure 5). Some distinct regions of no stain were observed between lamellae of the AF and within the NP. In Animals 2 and 3, the implant comprised approximately half of the AF ROI, however, in Animal 1, the measured implant area was appreciably less of the total AF ROI. The implant plate was clearly detached from the screws in Animal 1 (Supplementary Figure 6a) and was clearly retained by the screws in Animals 2 and 3 (Supplementary Figure 4 and Supplementary Figure 5).

### *Histomorphometry*

The morphology and composition of the tissue masses at the treatment site varied between the three Animals. Animals 1 and 3 exhibited masses outside of the treatment generally consisting of soft tissues (Supplementary Figure 4 and Supplementary Figure 5). However, Animal 2 demonstrated notable calcified tissue formations separated by a relatively dense region of soft tissue (Supplementary Figure 6). The ROIs of the tissue masses outside of the treated levels measured 95.9 mm^2^, 152.9 mm^2^, and 65.7 mm^2^ for Animals 1, 2, and 3, respectively. In these regions, all three Animals demonstrated blue soft tissue areas (11.8%, 26.9%, and 11.5%, respectively), green soft tissue areas (36.1%, 20.3%, and 24.8%, respectively), screw areas (9.1%, 9.7%, and 30.1%, respectively), and implant areas (25.9% 16.5%, and 23.2%, respectively). In Animal 2, the tissue mass comprised of 22.1% calcified tissue, and only 0.4% and 0.0% calcified tissue was detected for Animals 1 and 3, respectively. Additional, unidentified lesions were observed in the healthy AF and NP of Animal 2 in the micro-CT and histological results (Supplementary Figure 6c-d).

From the histomorphometric measurements (Supplementary Table 3 and Supplementary Table 4), blue and green soft tissues were the dominant stains in the healthy AF ROIs (combined blue and green soft tissue area was ≥ 82.6% in all Animals). Blue soft tissue area in the defect ROI of SRBS sections was lower than healthy range for all three Animals and was similarly lower for Animals 1 and 3 in the TBS sections. In both TBS and SRBS sections, the corresponding AF contralateral to the defect had blue soft tissue area within the healthy range for Animal 2 and Animal 3, but was also lower than the healthy range in Animal 1. The treatment ROI also exhibited distinctly lower blue soft tissue area than the corresponding healthy ranges in all three Animals in both SRBS and TBS sections, and the respective contralateral AF were all similar to the healthy range. Both defect and treatment ROIs were above the healthy range of green soft tissue, and the defect consistently demonstrated a greater level of green soft tissue as compared to the treatment. All contralateral AF ROIs had green soft tissue stain similar to the healthy AF. In the healthy NP, blue soft tissue was the dominant stain (≥ 82.1% ROI area for all Animals) for both SBRS and TBS sections. The NP in the defect levels exhibited areas of blue soft tissue similar to the healthy range for all Animals. Blue soft tissue in the NP of the treated levels was slightly lower than healthy NP for Animal 3 in the SRBS section, yet was similar in the TBS sections and for the other Animals. Small amounts of green soft tissue stain were observed in the healthy and defect-level NP (≤ 2.7% ROI area in all Animals), although the treated levels had slightly higher green soft tissue in the NP for Animal 1 and Animal 3. Measured bone area (red stain) was no more than 0.5% in all of the disc ROIs.

# Supplementary discussion

## *In vivo* ovine lumbar spine model

### *Radiographic imaging*

No clear changes in disc height or morphology of any disc level were observed between the 0-week and 12-week radiographs. Loss of disc height is a hallmark of degenerative disc disease(1) and, accordingly, would be a clear indicator if any of the interventions in the study led to major degenerative alteration of the disc. Measurement of the disc height via radiographs may have been limited by image resolution and orientation. Alternative imaging techniques, such as computed tomography (CT) or magnetic resonance imaging (MRI), may be able to enhance the precision of disc height measurements in future studies. The PCL scaffolds were not observable via plane radiographs and micro-CT, and the scaffolds were only observed via visual inspection and histological sectioning. Contrast agents could be utilized to improve visualization of the implant in *ex vivo* imaging, however, the implant may be challenging to capture with *in vivo* diagnostic imaging techniques. As a result, it may be difficult to assess the position and condition of the implant post-operatively and throughout the duration of healing. For example, the implant in this study that detached from the screws was not identified until *ex vivo* dissection of the spine was performed. Adding radiopaque markers, such as thin wire, to the implant or using magnetic resonance imaging may be considered in future studies to better image the implant *in vivo*.

### *Micro-computed tomography*

The largest bone volume measured by the micro-CT analyses was 2.04% (the left side of L_5_L_6_ disc in Animal 2); all other bone volumes were less than 1.0%. Further, imaging artefacts were observed in the scans which may have confounded measures of bone volume, particularly at the treated level which had artefact due to the metal screws used in the surgery. It is possible that the surgical intervention resulted in inflammation of the periosteal vertebral surfaces, resulting in the observed (small amount of) bone formation. However, overall, there was no clear evidence from the micro-CT results that indicated appreciable bone formation within any disc space in the study.

### *Histology*

Histological imaging of the healthy ovine IVDs clearly demonstrated the soft tissue composition and structure of the AF and NP. Sections stained with SRBS demonstrated a transition from intense and homogenous blue stain in the NP to green stain with a distinct lamellar organization in the outer AF, consistent with the well-known gradient from proteoglycan rich NP to the more organized collagenous outer AF.(2) Similar to SRBS sections, the TBS sections demonstrated a gradient from the strong proteoglycan staining in the NP to a lesser intensity in the outer AF. Some voids were observed in the histological sections and were most likely attributable to histological processing. The AF defects did not demonstrate the structure or composition of healthy AF at the 12-week time point. Because symptomatic re-herniation of the IVD is believed to be caused by loss of mechanical function of the AF(3,4), the AF defects exemplified a healing response that would lead to high risk of re-herniation. The treated AF also did not exhibit the structure or composition of the healthy AF and was shown to largely be composed of the implant. Although the treated AF did not present any major adverse tissue response as compared to the defect at the 12-week time point, the presence of the implant within the AF may provide an opportunity for long-term reconciliation of the structure and composition of healthy AF.

Bone formation within the disc space was of interest in this study due to the propensity for osteogenesis or osteophyte formation in the spine when the spinal biomechanics are altered. For example, fixation of the disc space is commonly used to generate bony growth in spinal fusion (5,6) and degenerative changes to the IVD are frequently associated with bony growth (e.g., osteophyte formation leading to spinal stenosis) (7) Accordingly, successful treatment for regeneration of the AF would inherently involve no bone formation in the disc space. Radiographic images did not indicate any appreciable increase in the density of the tissue in the disc space. However, histological imaging identified adverse tissue responses outside of the IVD. In particular, one animal developed an appreciable mass of calcified tissue around the implant with a small region of soft tissue in the plane of the IVD that had the appearance of a pseudoarthrosis or fibrocartilaginous callus (Supplementary Figure 6b). It is possible that this tissue formation was associated with the screw injury, periosteal disturbance, an inflammatory response to the implant, or an underlying pathology (because other abnormalities were observed in the healthy levels of the same Animal). However, there is insufficient evidence at this time to determine the cause of the bony growth. Regardless of the composition or cause of this tissue growth, it would be expected to have deleterious consequences with respect to the objective of arresting the long-term degeneration of the AF.

### *Histomorphometry*

In the histomorphometric analyses, the categorization of this gradient between green and blue soft tissue regions may have represented an arbitrary threshold of the two tissues (i.e., tissues stained with a blue-green color were classified as either blue or green). However, the same tissue classifications were used for all analyses and, therefore, the relative changes in proteoglycan and fibrous ECM composition were consistent in the study.

Supplementary Table 1. Disc height measurements (mean ± standard deviation, s.d., in mm) for the three study Animals at the 0-week (i.e., post-operative) and 12-week (i.e., post-sacrifice) time points.

| **Study** | **Level** | **Assignment** | **Disc height (mean ±s.d.)** | |
| --- | --- | --- | --- | --- |
|  |  |  | **0 weeks** | **12 weeks** |
| 1 | L_1_L_2_ | Sham | 3.9 ±1.3 | 3.2 ±1.4 |
|  | L_2_L_3_ | Healthy | 3.9 ±2.0 | 3.4 ±1.6 |
|  | L_3_L_4_ | Treatment | 3.5 ±1.4 | 3.3 ±1.5 |
|  | L_4_L_5_ | Healthy | 4.0 ±0.9 | 3.6 ±1.4 |
| 2 | L_1_L_2_ | Healthy | - | 3.8 ±1.7 |
|  | L_2_L_3_ | Sham | 4.6 ±1.5 | 4.3 ±1.8 |
|  | L_3_L_4_ | Healthy | 4.6 ±1.2 | 4.4 ±1.7 |
|  | L_4_L_5_ | Treatment | 4.4 ±1.0 | 4.0 ±1.2 |
|  | L_5_L_6_ | Healthy | 4.4 ±1.3 | 4.4 ±1.1 |
| 3 | L_1_L_2_ | Healthy | - | 3.5 ±2.2 |
|  | L_2_L_3_ | Sham | 4.7 ±1.5 | 3.5 ±1.7 |
|  | L_3_L_4_ | Healthy | 4.3 ±1.1 | 3.1 ±1.4 |
|  | L_4_L_5_ | Treatment | 4.2 ±1.4 | 3.5 ±1.6 |
|  | L_5_L_6_ | Healthy | 5.0 ±1.4 | 4.1 ±1.2 |

Supplementary Table 2. Micro-computed tomography bone volume analyses for all lumbar discs in the *in vivo* AF repair patch study. For the left and right halves of each disc, the total volume (TV), bone volume (BV), and percentage bone volume (BV/TV) are reported. The total measured disc volume (DV) is also reported for each disc.

| **Study** | **Level** | **Side** | **Assignment** | **TV** | **BV** | **TV/BV** | **DV** |
| --- | --- | --- | --- | --- | --- | --- | --- |
|  |  |  |  | **[mm^3^]** | **[mm^3^]** | **[%]** | **[mm^3^]** |
| 1 | L_1_L_2_ | Left | Sham - Sham | 407 | < 0.01 | < 0.01 | 882 |
|  |  | Right | Sham - Contralateral | 474 | < 0.01 | < 0.01 |  |
|  | L_2_L_3_ | Left | Healthy | 495 | < 0.01 | < 0.01 | 962 |
|  |  | Right | Healthy | 467 | < 0.01 | < 0.01 |  |
|  | L_3_L_4_ | Left | Treatment - Treatment | 531 | 0.07 | 0.01 | 962 |
|  |  | Right | Treatment - Contralateral | 431 | 0.04 | 0.01 |  |
|  | L_4_L_5_ | Left | Healthy | 646 | 0.07 | 0.01 | 1253 |
|  |  | Right | Healthy | 607 | < 0.01 | < 0.01 |  |
|  | L_5_L_6_ | Left | Healthy | 808 | 0.01 | < 0.01 | 1673 |
|  |  | Right | Healthy | 865 | < 0.01 | < 0.01 |  |
| 2 | L_1_L_2_ | Left | Healthy | 595 | 5.62 | 0.94 | 1163 |
|  |  | Right | Healthy | 567 | 0.38 | 0.07 |  |
|  | L_2_L_3_ | Left | Sham - Sham | 549 | 0.25 | 0.05 | 1033 |
|  |  | Right | Sham - Contralateral | 483 | 0.06 | 0.01 |  |
|  | L_3_L_4_ | Left | Healthy | 626 | 0.05 | 0.01 | 1266 |
|  |  | Right | Healthy | 640 | < 0.01 | < 0.01 |  |
|  | L_4_L_5_ | Left | Treatment - Treatment | 460 | 0.05 | 0.01 | 916 |
|  |  | Right | Treatment - Contralateral | 456 | < 0.01 | < 0.01 |  |
|  | L_5_L_6_ | Left | Healthy | 621 | 12.69 | 2.04 | 1266 |
|  |  | Right | Healthy | 645 | 2.98 | 0.46 |  |
| 3 | L_1_L_2_ | Left | Healthy | 496 | 0.14 | 0.03 | 947 |
|  |  | Right | Healthy | 450 | 0.01 | < 0.01 |  |
|  | L_2_L_3_ | Left | Sham - Sham | 486 | 0.04 | 0.01 | 892 |
|  |  | Right | Sham - Contralateral | 406 | 0.01 | < 0.01 |  |
|  | L_3_L_4_ | Left | Healthy | 601 | < 0.01 | < 0.01 | 1113 |
|  |  | Right | Healthy | 512 | 0.01 | < 0.01 |  |
|  | L_4_L_5_ | Left | Treatment - Treatment | 552 | 0.62 | 0.11 | 1165 |
|  |  | Right | Treatment - Contralateral | 613 | 0.23 | 0.04 |  |
|  | L_5_L_6_ | Left | Healthy | 886 | 3.82 | 0.43 | 1674 |
|  |  | Right | Healthy | 788 | 2.42 | 0.31 |  |

Supplementary Table 3. Summary of histomorphometric measurements for sections of all discs in the *in vivo* study stained with SRBS with Van Gieson’s Counterstain. Measurements for the healthy conditions are presented as the range of all corresponding ROI in the healthy levels. All other measurements are from a single ROI. The healthy contralateral (CL) AF measurements are shown independently from the defect/treatment measurements. Blue soft tissue stain was indicative of proteoglycan-rich tissue and green soft tissue stain was indicative of fibrous tissue.

| **Animal** | **ROI** | **Condition** | **ROI area [mm^2^]** | **Blue soft tissue area [%]** | **Green soft tissue area [%]** | **Bone area [%]** | **Implant area [%]** |
| --- | --- | --- | --- | --- | --- | --- | --- |
| 1 | AF | Healthy | 10.3 - 13.2 | 34.9 - 74.9 | 16.9 - 47.7 | 0.0 - 0.3 | - |
|  |  | Defect | 10.9 | 23.7 | 69.4 | 0.0 | - |
|  |  | Defect (CL) | 8.7 | 29.1 | 50.8 | 0.0 | - |
|  |  | Treatment | 16.5 | 1.7 | 65.6 | 0.0 | 17.7 |
|  |  | Treatment (CL) | 8.8 | 67.2 | 24.8 | 0.5 | - |
|  | NP | Healthy | 20.0 - 61.7 | 82.1 - 94.5 | 0.0 - 2.7 | 0.0 - 0.1 | - |
|  |  | Defect | 26.9 | 97.9 | 0.8 | 0.0 | - |
|  |  | Treatment | 28.5 | 92.9 | 6.8 | 0.0 | - |
| 2 | AF | Healthy | 7.5 - 13.5 | 51.6 - 72.3 | 17.4 - 36.2 | 0.0 - 0.2 | - |
|  |  | Defect | 18.3 | 28.3 | 69.0 | 0.2 | - |
|  |  | Defect (CL) | 10.0 | 58.9 | 29.8 | 0.2 | - |
|  |  | Treatment | 18.1 | 28.6 | 19.7 | 0.0 | 46.8 |
|  |  | Treatment (CL) | 12.3 | 69.8 | 26.4 | 0.0 | - |
|  | NP | Healthy | 36.0 - 64.4 | 97.0 - 99.8 | 0.2 - 2.2 | 0.0 | - |
|  |  | Defect | 33.7 | 99.3 | 0.6 | 0.1 | - |
|  |  | Treatment | 42.5 | 99.3 | 0.3 | 0.0 | - |
| 3 | AF | Healthy | 9.0 - 14.5 | 44.8 - 64.9 | 29.9 - 46.8 | 0.0 - 0.4 | - |
|  |  | Defect | 14.2 | 25.1 | 56.5 | 0.1 | - |
|  |  | Defect (CL) | 9.9 | 49.0 | 42.4 | 0.1 | - |
|  |  | Treatment | 14.8 | 14.0 | 32.8 | 0.0 | 46.3 |
|  |  | Treatment (CL) | 11.3 | 67.0 | 31.9 | 0.1 | - |
|  | NP | Healthy | 35.4 - 71.1 | 85.7 - 100.0 | 0.0 - 1.6 | 0.0 | - |
|  |  | Defect | 38.0 | 99.9 | 0.1 | 0.0 | - |
|  |  | Treatment | 63.9 | 79.2 | 3.6 | 0.0 | - |

Supplementary Table 4. Summary of histomorphometric measurements for sections of all discs in the *in vivo* study stained with TBS. Measurements for the healthy conditions are presented as the range of all corresponding ROI in the healthy levels. All other measurements are from a single ROI. The healthy contralateral AF measurements are shown independently from the defect/treatment measurements. Blue soft tissue stain was indicative of proteoglycan-rich tissue.

| **Animal** | **ROI** | **Condition** | **ROI area [mm^2^]** | **Soft tissue (blue) area [%]** | **Implant area [%]** |
| --- | --- | --- | --- | --- | --- |
| 1 | AF | Healthy | 11.3 - 58.9 | 90.4 - 92.2 | - |
|  |  | Defect | 46.8 | 37.6 | - |
|  |  | Defect (contralateral) | 34.3 | 77.7 | - |
|  |  | Treatment | 67.5 | 63.7 | 19.7 |
|  |  | Treatment (contralateral) | 37.5 | 89.1 | - |
|  | NP | Healthy | 95.0 - 222.0 | 94.2 - 94.3 | - |
|  |  | Defect | 99.1 | 92.1 | - |
|  |  | Treatment | 109.8 | 99.3 | - |
| 2 | AF | Healthy | 10.9 - 42.1 | 83.0 - 98.0 | - |
|  |  | Defect | 64.5 | 94.3 | - |
|  |  | Defect (contralateral) | 35.7 | 92.7 | - |
|  |  | Treatment | 17.4 | 37.5 | 55.1 |
|  |  | Treatment (contralateral) | 10.5 | 93.0 | - |
|  | NP | Healthy | 41.6 - 236.2 | 97.8 - 100.0 | - |
|  |  | Defect | 140.6 | 100.0 | - |
|  |  | Treatment | 43.1 | 97.6 | - |
| 3 | AF | Healthy | 9.3 - 15.9 | 83.3 - 94.0 | - |
|  |  | Defect | 15.0 | 66.2 | - |
|  |  | Defect (contralateral) | 7.4 | 87.9 | - |
|  |  | Treatment | 16.8 | 40.8 | 43.8 |
|  |  | Treatment (contralateral) | 12.7 | 90.9 | - |
|  | NP | Healthy | 45.3 - 74.8 | 83.3 - 100.0 |  |
|  |  | Defect | 42.7 | 83.4 | - |
|  |  | Treatment | 64.5 | 91.5 | - |


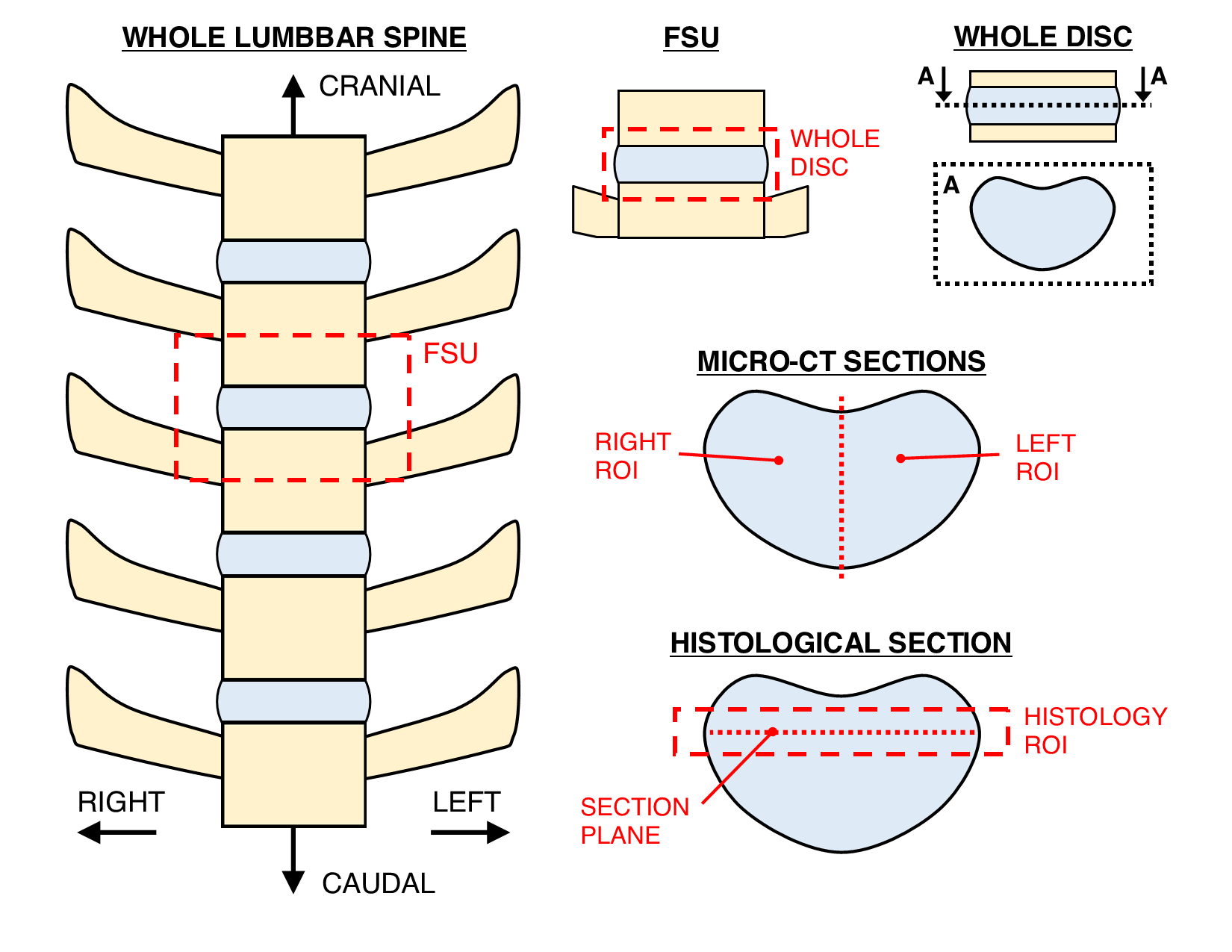


**Supplementary Figure 1.** Diagram of a whole ovine lumbar spine showing the functional spinal unit (FSU) for biomechanical testing, the whole disc section for micro-computed tomography (micro-CT), and the partial disc section for histology. Red dashed lines indicate physical sectioning of the samples. For bone volume analysis of the micro-CT scan, left and right ROIs were defined by divided the IVD centrally in the sagittal plane. The section plane for histological images is also shown (oriented in the coronal plane).


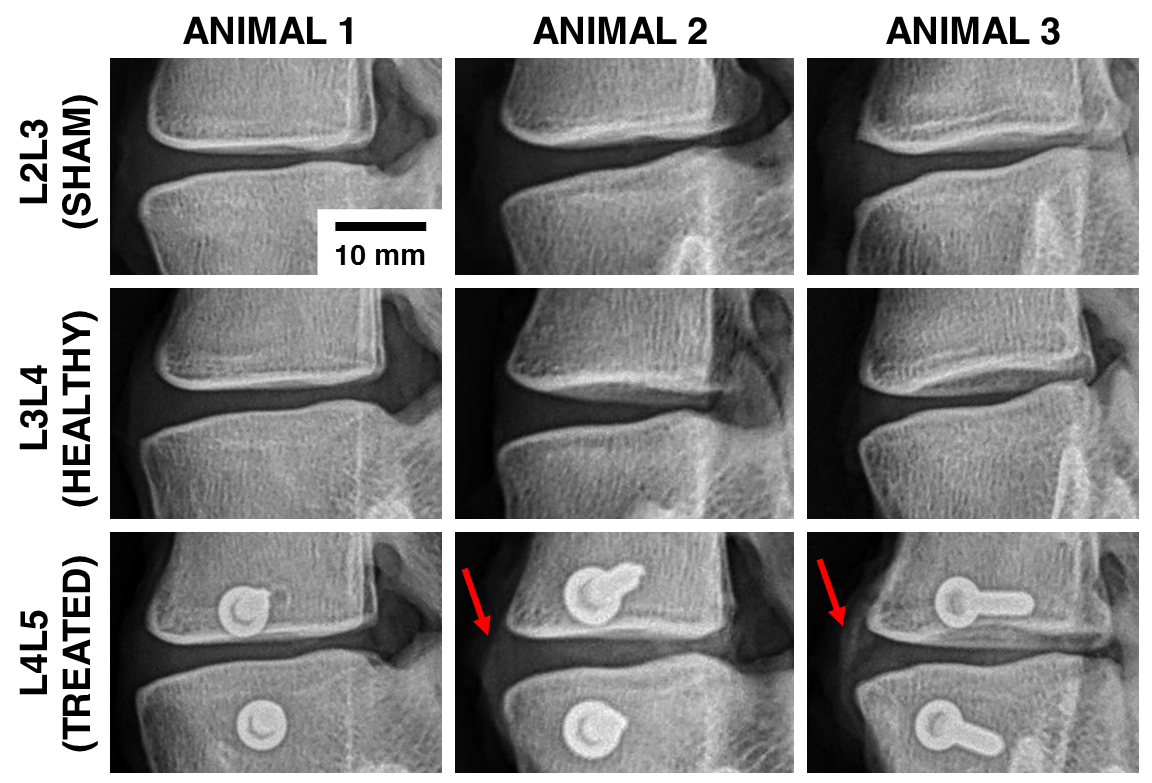


**Supplementary Figure 2.** Radiographic images of three intervertebral disc spaces (sham, healthy, and treated) for the three experimental animals at the 12-week time point. The metal screws used to fix the implants are clearly visible in the treated levels. As compared to the corresponding healthy and sham levels, regions of slightly increased radiopacity were observed surrounding the treated levels of Animals 2 and 3 (red arrows).


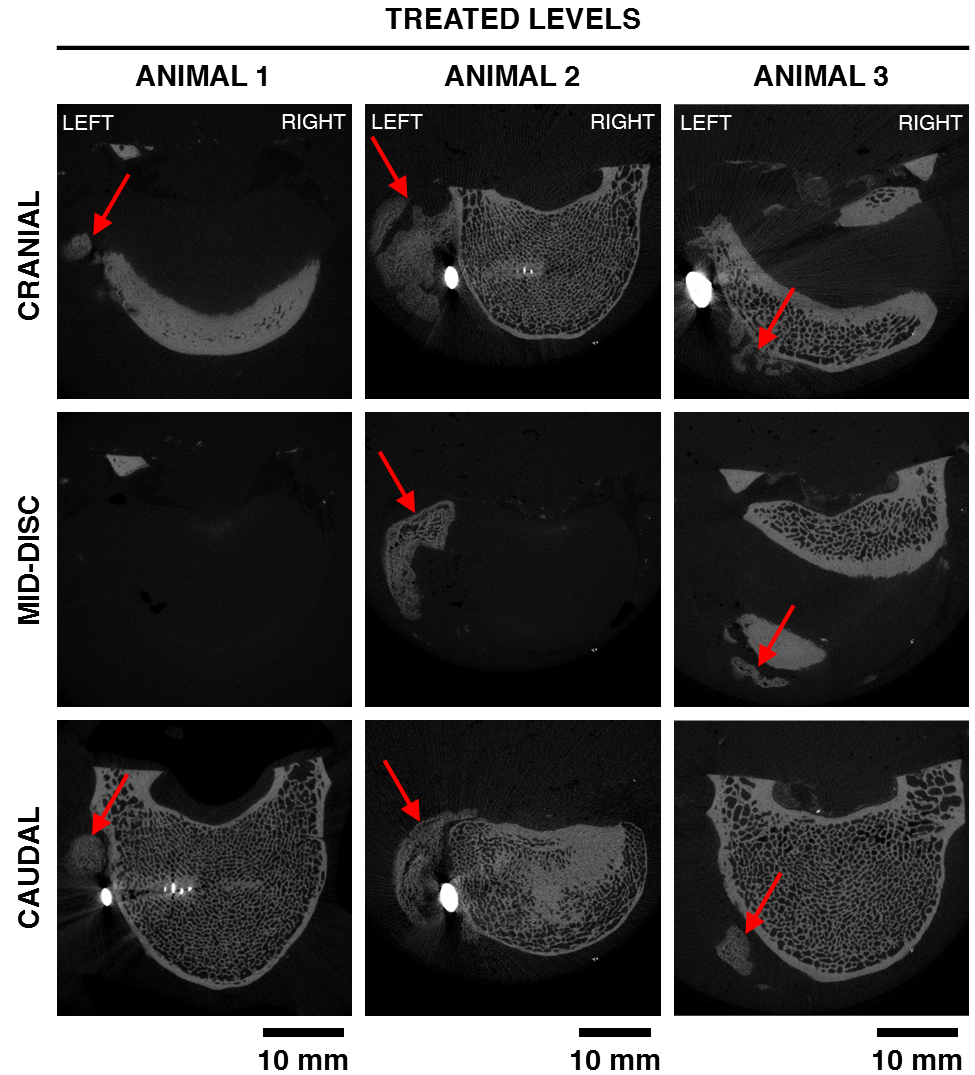


**Supplementary Figure 3.** Micro-computed tomography images in the transverse plane of the treated levels for all three studies. Example images are shown at the mid-disc level and at the approximate level of the screws in the cranial and caudal directions. The screws are visible as bright white objects and scanning artefacts associated with the screws are visible as lines propagating from the screw location. Extraneous dense tissue masses are indicated with red arrows.

**
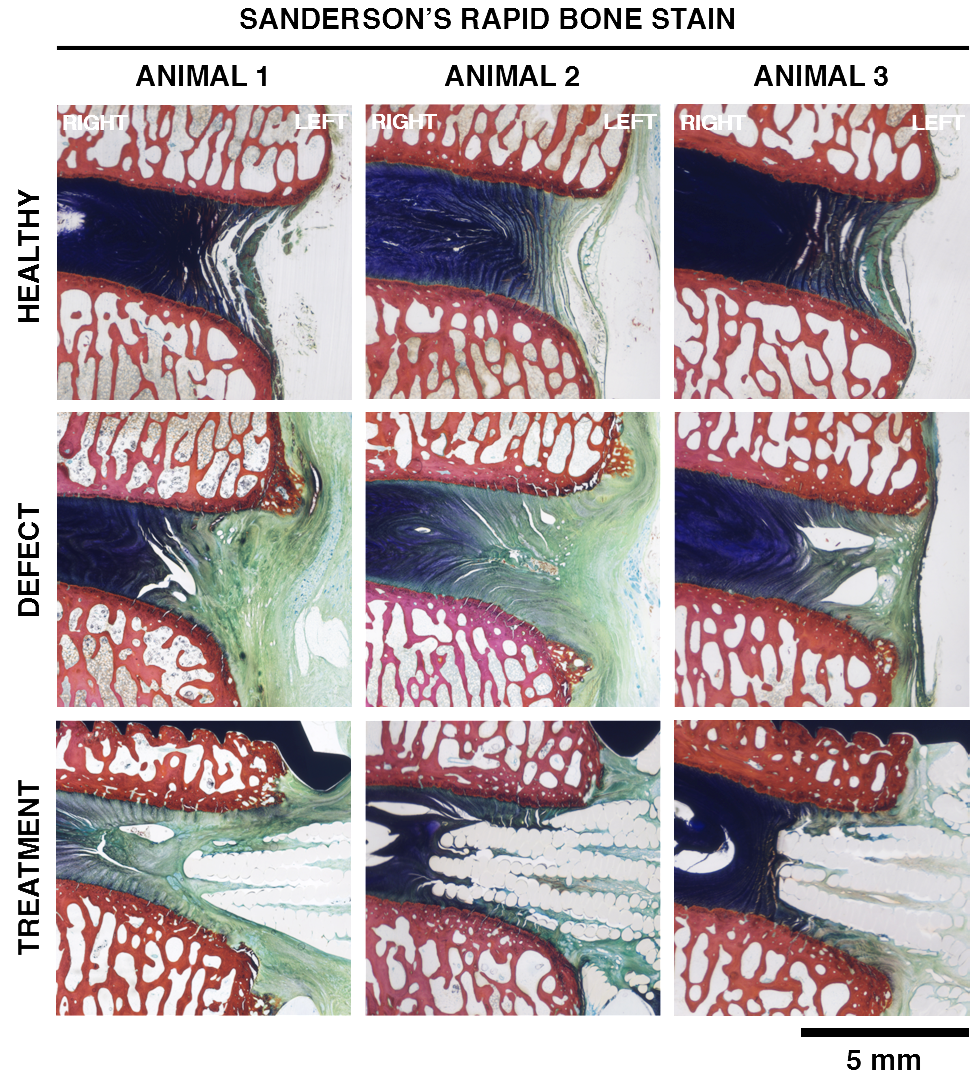
**

**Supplementary Figure 4.** Histological sections of the left AF ROI stained with Sanderson’s Rapid Bone Stain. For all three animals in the study, the healthy AF tissue demonstrated a distinct lamellar structure and composition which were not restored in the defect levels.


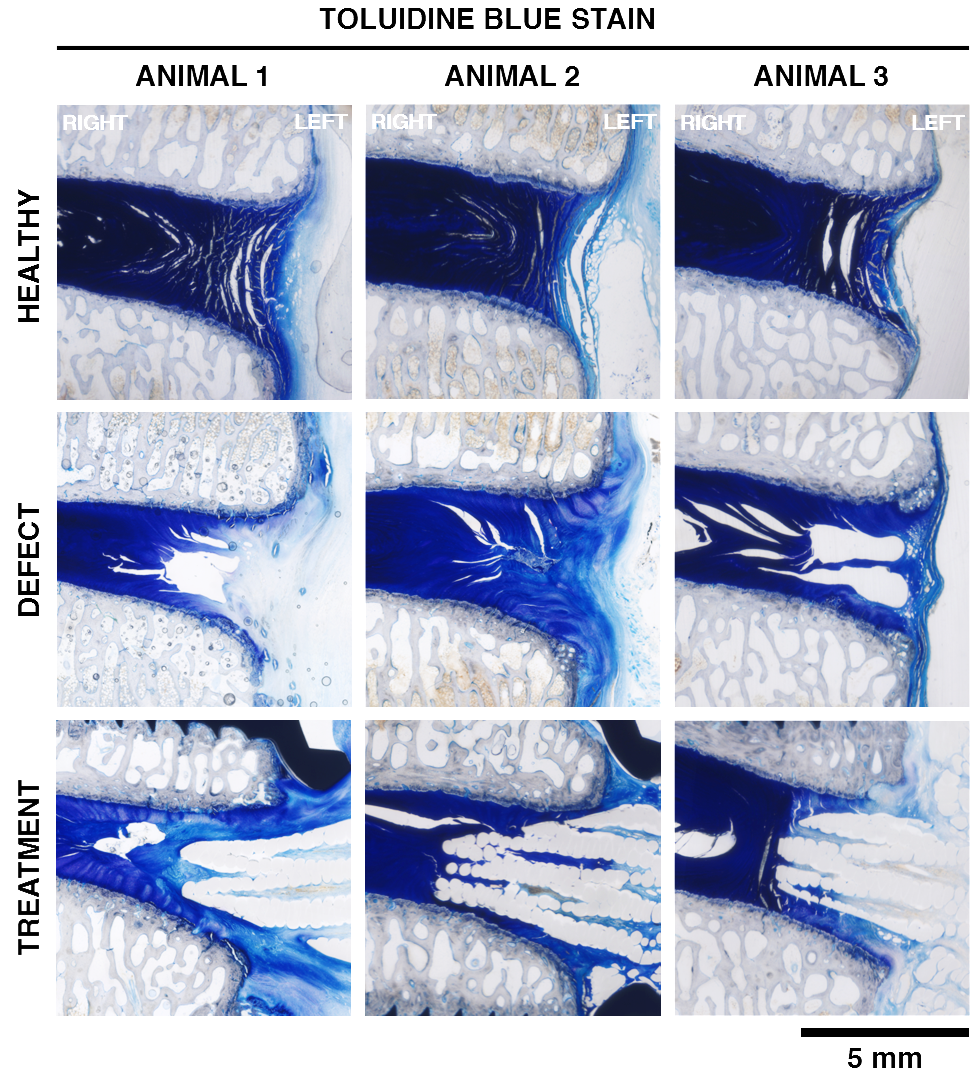


**Supplementary Figure 5.** Histological sections of the left AF ROI stained with Toluidine Blue Stain. Similar to Supplementary Figure 4, the distinct structure and composition of the healthy AF was not restored in the defect level for all three animals in the study.


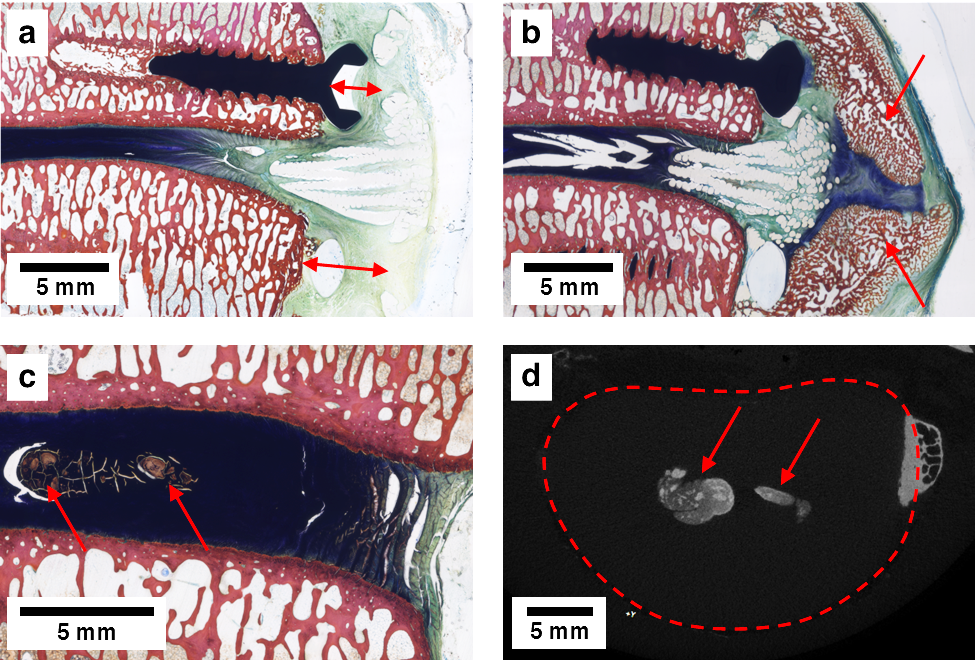


**Supplementary Figure 6.** Notable features of the *in vivo* animal study: (a) histological image of the treated level of Animal 1 showing the displacement of the implant (red arrows); (b) histological image of the treated level of Animal 2 showing the calcified tissue growth (red arrows); (c) histological image of the untreated L_5_L_6_ disc of Animal 2 showing unidentified lesions in the NP (red arrows); and (d) micro-computed tomography image of the untreated L_5_L_6_ disc of Animal 2 showing the unidentified lesions (red arrows) within the disc space (red dashed line).
